# Relationship between Heat-Labile Enterotoxin Secretion Capacity and Virulence in Wild Type Porcine-Origin Enterotoxigenic *Escherichia coli* Strains

**DOI:** 10.1371/journal.pone.0117663

**Published:** 2015-03-13

**Authors:** Prageeth Wijemanne, Jun Xing, Emil M. Berberov, David B. Marx, David H. Francis, Rodney A. Moxley

**Affiliations:** 1 School of Veterinary Medicine and Biomedical Sciences, University of Nebraska-Lincoln, Lincoln, Nebraska, United States of America; 2 Canada Immunoassay Development, Windsor, Ontario, Canada; 3 Vaccine and Infectious Disease Organization-International Vaccine Centre, University of Saskatchewan, Saskatoon, Saskatchewan, Canada; 4 Department of Statistics, University of Nebraska-Lincoln, Lincoln, Nebraska, United States of America; 5 Department of Veterinary Science, South Dakota State University, Brookings, South Dakota, United States of America; The Ohio State University, UNITED STATES

## Abstract

Heat-labile enterotoxin (LT) is an important virulence factor secreted by some strains of enterotoxigenic *Escherichia coli* (ETEC). The prototypic human-origin strain H10407 secretes LT via a type II secretion system (T2SS). We sought to determine the relationship between the capacity to secrete LT and virulence in porcine-origin wild type (WT) ETEC strains. Sixteen WT ETEC strains isolated from cases of severe diarrheal disease were analyzed by GM1ganglioside enzyme-linked immunosorbent assay to measure LT concentrations in culture supernatants. All strains had detectable LT in supernatants by 2 h of culture and 1 strain, which was particularly virulent in gnotobiotic piglets (3030-2), had the highest LT secretion level all porcine-origin WT strains tested (P<0.05). The level of LT secretion (concentration in supernatants at 6-h culture) explained 92% of the variation in time-to-a-moribund-condition (R^2^ = 0.92, *P*<0.0001) in gnotobiotic piglets inoculated with either strain 3030-2, or an ETEC strain of lesser virulence (2534-86), or a non-enterotoxigenic WT strain (G58-1). All 16 porcine ETEC strains were positive by PCR analysis for the T2SS genes, *gspD* and *gspK*, and bioinformatic analysis of 4 porcine-origin strains for which complete genomic sequences were available revealed a T2SS with a high degree of homology to that of H10407. Maximum Likelihood phylogenetic trees constructed using T2SS genes *gspC*, *gspD*, *gspE* and homologs showed that strains 2534-86 and 3030-2 clustered together in the same clade with other porcine-origin ETEC strains in the database, UMNK88 and UMN18. Protein modeling of the ATPase gene (*gspE*) further revealed a direct relationship between the predicted ATP-binding capacities and LT secretion levels as follows: H10407, -8.8 kcal/mol and 199 ng/ml; 3030-2, -8.6 kcal/mol and 133 ng/ml; and 2534-86, -8.5 kcal/mol and 80 ng/ml. This study demonstrated a direct relationship between predicted ATP-binding capacity of GspE and LT secretion, and between the latter and virulence.

## Introduction

Enterotoxigenic *Escherichia coli* (ETEC) strains are important causes of diarrhea among travelers and children <5 years old living in developing countries [35], and in addition, are economically important pathogens of pigs and cattle [27,37]. ETEC infections are especially severe among young swine, causing illness and deaths of nursing and post-weaned piglets [13]. In swine, the most common and severe ETEC infections are caused by strains that express F4 (K88) fimbria [4]. These strains usually produce two major enterotoxins that cause net fluid loss and diarrhea, viz., heat-labile enterotoxin (LT) and heat-stable enterotoxin-b (STb) [22,24]. Some strains also may produce heat stable enterotoxin-a (STa) or enteroaggregative *E*. *coli* heat-stable enterotoxin 1 [4].

Tauschek et al. [34] discovered a type II secretion system (T2SS) in the human prototypic ETEC strain H10407 similar to the one in *Vibrio cholerae*, and demonstrated that it is functional and necessary for secretion of LT in ETEC. Both Tauschek et al. [34] and Lasaro et al. [21] demonstrated the presence *gspD* and *gspK* in ETEC strains from human patients and from this observation inferred that the T2SS is highly conserved among ETEC. However, to our knowledge, no porcine-origin ETEC strains have been tested for these genes and among human-origin strains only the H10407 T2SS sequence has been analyzed [34].

Lasaro et al. [21] hypothesized that strain-specific differences in production and secretion of LT by human-origin ETEC correlated with symptoms induced in vivo. Although these authors demonstrated that the secretion levels, in contrast to the total amounts of LT produced were correlated with volumes of fluid accumulation in ligated rabbit ileal loops, the authors were not able to demonstrate a relationship between LT secretion and clinical symptoms in human patients. In that study, the clinical data was limited to presence or absence of diarrhea, and whether the affected children were co-infected with other pathogens was not reported.

In the present study, we sought to test the hypothesis that LT secretion is correlated with virulence using wild-type porcine-origin ETEC strains for which we had clinical and pathological records of natural cases of disease, and conclusive evidence that ETEC was the sole cause of disease. In addition, for two of these ETEC strains which differed in virulence and a non-enterotoxigenic wild-type porcine-origin control strain, we conducted experimental gnotobiotic pig inoculations and also had genomic sequence data available. Hence, using these strains, we assessed the relationship between virulence and LT secretion, and also compared the sequences of T2SS genes by bioinformatics analysis.

## Materials and Methods

### Strains

The strains used in this study are shown in S1 Table. Included among these were 16 porcine-origin wild type (WT) LT^+^ STb^+^ ETEC strains from cases of severe diarrheal disease or sudden death, most with lesions of hemorrhagic enteritis [10,14,25], human WT ETEC strain H10407 (O78:K80:H11, CFA/1^+^, LT^+^, STp^+^, STh^+^) [12], and non-pathogenic LT^-^ STb^-^ *E*. *coli* control strains [18,36]. All strains were confirmed to have the appropriate LT (*eltAB*) and STb (*estB*) gene content by PCR using primers and methods as previously described [4].

### Media and Growth Conditions

In order to properly assess levels of LT secretion, we needed to use optimized culture conditions, as this has been reported to have a significant effect on LT production and release in previous studies. Casamino Acids-yeast extract (CAYE) medium-Mundell (CAYE-M) [26,29] was prepared with Bacto Casamino Acids (2.0%) and Difco yeast extract (0.6%), and glucose at a final concentration of 0.25%, and adjusted to a final pH of 8.5.

Our first experiment aimed to determine whether porcine-origin WT ETEC strains could secrete LT. This experiment utilized prototype human-origin ETEC strain H10407 as a positive control;, test strains 2534–86 and its isogenic derivatives [MUN297(LT^+^), MUN299 (LT^-^), MUN300 (LT^-^), and MUN301 (LT^+^)]; negative control K-12 strain DH5α; positive control strain MUN302 (a DH5α-based LT^+^ clone); and WT porcine-origin negative controls (LT^-^ strains 1836–2 and G58–1). For this experiment, starter cultures were grown overnight in 15 ml of CAYE-M in a 125 ml conical flask at 37°C and 225 rpm. After overnight incubation, a 1:100 dilution of each starter culture was inoculated into fresh medium of the same kind using the same flask-to-medium ratio (8.3:1), and incubated for 18 h at 37°C and 225 rpm. Samples were then obtained for preparation of supernatants and periplasmic extracts for LT determination.

A second experiment as a test of LT secretion attempted to rule out bacterial cell lysis as a contributor to LT presence in the culture supernatant. This experiment utilized porcine-origin LT^+^ WT strain 2534–86, 2 isogenic derivatives of this strain (LT^+^ MUN298 and LT^-^ MUN299), and human-origin prototype strain H10407, with strains grown in CAYE-M. For this experiment, starter and experimental cultures were prepared in CAYE-M as described for the first experiment, but samples were collected at 4, 8, 12 and 24 h PI. To confirm that growth rates in CAYE-M among strains did not vary significantly, results which could confound the measured LT concentrations, growth curves on all strains were conducted with samples obtained at 0, 4, 8, 12 and 24 h. On aliquots of these samples, the OD_600_, colony-forming units (CFU)/ ml, and pH values were determined. CFU/ml were determined by serial 10-fold dilution in phosphate-buffered saline (pH 7.4, 0.1 *M*; PBS) and plating on LB agar.

A third experiment tested the capacity for 16 WT LT^+^ porcine strains isolated from cases of severe disease to secrete LT (S1 Table), and was conducted using the same protocol as that used in the second experiment, except that samples were collected at 2, 4, 6 and 18 h of culture.

For each of the 3 main experiments, 3 independent replicate experiments were conducted on different days using new cultures.

### Supernatant and Periplasmic Extract Preparation

To prepare cell-free supernatant, 1-ml aliquots of bacterial cultures were collected, centrifuged at 2,150 × *g* for 10 min, and passed through a 0.2 μm filter. To prepare periplasmic extracts, the corresponding cell pellets from the same aliquots were given 3 series of suspensions in 1 ml of PBS and centrifugations at 2,150 × *g* for 10 min. The final re-suspension was in 1 ml of 2 mg/ml solution of polymyxin B in PBS for 30 min in a 37°C water bath; this was centrifuged at 2,150 × *g* for 10 min, 0.2 μm-filtered and the liquid fraction was obtained as the periplasmic extract. For each sample of culture supernatant and periplasmic extract, a lack of contamination by live cells of the strain of origin was confirmed by culturing an aliquot overnight in LB broth and on LB agar at 37°C. To test whether LT was present in culture supernatants as a result of secretion, bacterial lysis or both during culture, aliquots of culture supernatants at 4, 6, 12, 18, and 24 h and periplasmic extracts at 2, 4, and 6 h of culture were mixed with equal volumes of *p*-nitrophenyl phosphate (PNPP, Sigma), the substrate for alkaline phosphatase, which is normally present in high content in the periplasmic space in intact *E*. *coli* cells [34]. Mixtures of sample and PNPP in 0.2 M Tris buffer in 96-well plates were incubated at room temperature for 30 min. At that time, 25 μl of 5.0 N NaOH was added to stop the reaction, and the OD_405_ was measured.

### Assays for LT Production and Secretion

The concentrations of LT in the periplasmic extracts and culture supernatant were determined by GM1- ganglioside enzyme-linked immunosorbent assay (GM1-ELISA) using methods previously described [29,34] with the following modifications. All washings were done with 1% Tween 20 in PBS. The primary antibody was a rabbit anti-cholera toxin IgG (Sigma) at a 1:2000 dilution, and the secondary antibody was alkaline phosphatase-conjugated goat anti-rabbit IgG (Sigma) at a 1:16,000 dilution in the first experiment, and at a 1:8000 dilution in the second and third experiments. Incubations of 60 min at 37°C were given after the addition of each antibody. The substrate was PNPP (Sigma) in 0.2 M Tris buffer, and after its addition, plates were incubated for 30 min at room temperature and OD measured at 405 nm. Standard curves were generated in each assay plate by using 2-fold serial dilutions of purified LT (List Biological Laboratories, Product No. 165B) at a starting concentration of 400 ng/ml. Regression analysis (R^2^>0.97) was used to generate a standard curve for determination of LT concentrations in the test samples.

Culture supernatants of MUN297, MUN299, MUN300, MUN301, MUN302, H10407, 1836–2, G58–1, DH5α, 2534–86, WAM2317, and 3030–2 were tested in Y1 adrenal cell assays to confirm that LT biological activity was present or absent in samples from LT^+^ and LT^-^ strains, respectively. Y1 adrenal cell assays were conducted by the methods of Sack and Sack [30] with minor modifications. Y1 adrenal cells from ATCC (CCL-79) were grown in F-12K medium (ATCC) supplemented with 15% horse serum (ATCC) and 2.5% fetal bovine serum (Sigma). For the LT assays, 100 µl of culture supernatants diluted 1:10 were added onto Y1 cell monolayers in duplicate wells in the initial rows of 96-well microtiter plates, and then were serially 2-fold diluted in the plates to 1:1,280. To the second row of inoculated wells for each sample, 100 μl of rabbit anti-cholera toxin IgG (Sigma) at a 1:100 dilution in PBS was added. At 4 and 24 h post-inoculation (PI), all wells were observed with an inverted microscope; a well was considered positive if >50% of the cells in the respective monolayer were rounded. The titer was the highest dilution at which an inoculated well was positive and the corresponding well at the same dilution containing inoculum plus anti-toxin serum was negative.

### Gnotobiotic Piglet Experiments

Twelve F4 receptor-positive gnotobiotic piglets were inoculated at 7–9 days of age with strains 3030–2 (n = 5), 2534–86 (n = 5), or G58–1 (n = 2) using methods previously described [4] with minor modifications to determine whether these strains differed significantly in the number of h PI for a moribund condition to occur. Experiments were approved by the University of Nebraska Institutional Animal Care and Use Committee. In these experiments, piglets were checked at 1–4 h intervals for depression, lethargy, diarrhea, and dehydration, and were euthanized when moribund, or at 96 h PI if this condition did not occur. The h PI for a moribund condition to occur was used for linear regression analysis along with LT secretion values (ng/ml) for the same strains. Piglets were necropsied immediately after euthanasia and tissues were collected by aseptic technique and processed for culture and histopathology as previously described [4]. Blood samples obtained at necropsy also were tested for endotoxin activity using the *Limulus* Amebocyte Lysate QCL-1000 assay (Lonza Walkersville, Walkersville, MD) following the manufacturer’s instructions.

### Analysis of Porcine ETEC Strains for Type II Secretion System

Using primers and conditions as described by Tauschek et al. [34], PCR for *gspD* and *gspK* was conducted to determine the prevalence of the T2SS in the porcine ETEC strains listed in S1 Table. As a further analysis of the T2SS, using Geneious 6.1.3, nucleotide and amino acid sequences of 3 porcine strains, viz., 2534–86 (Accession no. AFDS01000066), 3030–2 (Accession no. AFDT01000052), and G58–1 (AFDX01000001) were aligned with that of H10407 (Accession no. AY056599) and *Vibrio cholerae* TRH7000 (Accession no. L33796). Furthermore, they were aligned with 2 other porcine ETEC in the NCBI Database, viz., UMNK88 (CP002729) and UMNF18 (AGTD00000000) [31]. To construct phylogenetic trees and protein models, the following were used in addition to the ETEC strains; *Aeromonas hydrophila* AL09–71 (CP007566), *Aeromonas salmonicida* 449 (CP000644), *Aeromonas veronii* B565 (CP002607), *Burkholderia mallei* ATCC 10399 (CH899680), *Burkholderia pseudomallei* K96243 (BX571965), *Dickeya chrysanthemi* (L02214), *Dickeya dadantii* 3937 (CP002038), *Dickeya zeae* Ech1591 (CP001655), *Erwinia pyrifoliae* Ejp617 (CP002124), *Escherichia coli* BW2952 (CP001396), *Escherichia coli* CE10 (CP001396), *Escherichia coli* EC958 (HG941718), *Escherichia coli* LF82 (CU651637), *Escherichia coli* MG1655 (U00096), *Escherichia coli* Nissle 1917 (CP007799), *Escherichia coli* NRG 857C (CP001855), *Escherichia coli* W3110 (AP009048), *Klebsiella oxytoca* HKOLP1 (CP004887), *Klebsiella pneumoniae* ATCC BAA-2146 (CP006659), *Legionella longbeachae* D-4968 (ACZG01000001), *Pectobacterium carotovorum* (X70049) *Pseudomonas aeruginosa* PA1 (CP004054), *Pseudomonas putida* H8234 (CP005976), *Shewanella amazonensis* SB2B (CP000507), *Shewanella loihica* PV-4 (CP000606), *Shewanella putrefaciens* 200 (CP002457), and *Vibrio vulnificus* (CP002469). Protein models for all the strains were created using the SWISS-MODEL server platform (swissmodel.expasy.org) using *V*. *cholerae*, *V*. *vulnificus* and *E*. *coli* T2SS protein crystal structures in the Protein Data Bank. PyRx Python Prescription 0.8 was used to analyze the binding capacities of all the ATPases using models generated by the SWISS-MODEL server as the substrate and ATP as the ligand. Phylogenetic analyses of *gspC*, *gspD*, *gspE* and homologs were conducted using MEGA6 [33]. The evolutionary history was inferred by using the Maximum Likelihood method based on the Tamura-Nei model [32]. Initial tree(s) for the heuristic search were obtained automatically by applying Neighbor-Joining and BioNJ algorithms to a matrix of pairwise distances estimated using the Maximum Composite Likelihood (MCL) approach, and then selecting the topology with superior log likelihood value. Trees were drawn to scale, with branch lengths measured in the number of substitutions per site. Codon positions included were 1st+2nd+3rd+noncoding, with all positions containing gaps and missing data eliminated. *Escherichia coli* ATCC 25922 16S rRNA (DQ360844.1:86278349) was used as an out group. For protein alignment studies, PyMOL Molecular Graphics System version 1.3 was used.

### Statistical Analyses

Statistical Analysis System (SAS, Version 9.4, Cary, NC) software was used to analyze the data for effect of strain on secretion, and strain on virulence. A test for a linear association between LT secretion and h-to-a-moribund-condition was run for each of the 2 times at which culture supernatant LT concentrations (ng/ml) were measured (6 and 18 h), and tested for lack of fit. The coefficient of determination (R^2^) was calculated for each regression analysis. In addition, the linear regressions for each time were compared to see if the slopes were different. Analysis of variance for each time was conducted for comparisons between human and porcine strains as well as among the WT porcine strains and separated using a protected least significant difference test. Calculated *P* values of < 0.05 were considered significant.

## Results

### LT Secretion by Porcine ETEC Strains

As an initial test of the capacity for porcine-origin WT strains to secrete LT, supernatants from 18-h cultures of 2534–86, 3030–2, and derivatives of 2534–86 grown in CAYE-M medium were analyzed by GM1-ELISA and the Y-1 adrenal cell assay. Culture supernatants of all ETEC strains with detectable GM1-ELISA activity also had activity in the Y1 assay after 24 h PI, whereas non-enterotoxigenic *E*. *coli* strains lacked activity in both assays (S2 Table). Y1 activity was not present at 4 h PI with any of the strains, suggesting that activity seen at 24 h PI was not due to other toxic effects. Cell rounding was inhibited by incubation of culture supernatants with anti-CT antiserum prior to inoculation. A lack of contamination of each of the culture supernatants with the respective strains under growth also was confirmed by LB broth and agar cultures. Although these results confirmed secretion of biologically active LT, the results were largely qualitative, and involved the testing of only 2 porcine-origin WT ETEC strains. The secondary antibodies used in the GM1-ELISA had not been optimized at the time this experiment was done, so results were interpreted as positive or negative as reported in S2 Table, and results were only semi-quantitative in the case of the Y1 assay. In addition, use of 18-h cultures raised the question that some of the LT activity in the culture supernatant could have resulted from bacterial lysis. To confirm that strain 2534–86 secreted LT and to compare secreted levels with that of H10407, 2534–86, isogenic derivatives MUN298 (LT^+^) and MUN299 (LT^-^), and H10407 grown in CAYE-M were sampled at 4, 8, 12, and 24 h, and LT concentrations in culture supernatants and periplasmic extracts determined with an optimized GM1-ELISA. LT concentrations in the culture supernatant were significantly higher for H10407 (*P*<0.05), whereas no significant difference was detected between 2534–86 and MUN298, and MUN299 was confirmed to have no detectable LT activity (S1 Fig). Periplasmic LT concentrations were also highest for H10407 (*P*<0.05), and peaked at 4 h of culture for this and the other 2 LT^+^ strains (S2 Fig). Growth curves (S3 Fig) demonstrated that the CFU/ml did not significantly differ among strains, and all exhibited the same drop in pH at 4 h and increase in pH thereafter, which reflected utilization of glucose through aerobic fermentation, the production and accumulation of organic acids, and afterward their utilization as energy sources.

**Fig 1:**
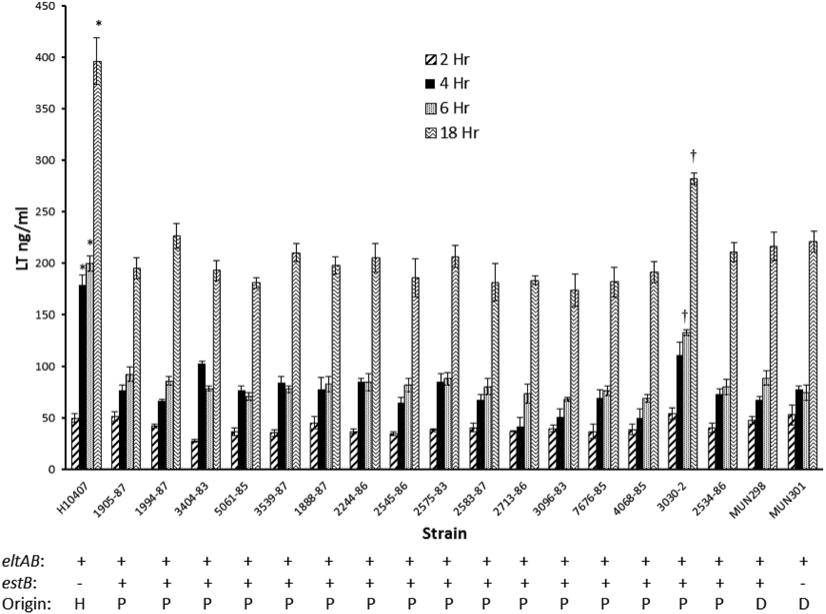
Heat-labile enterotoxin (LT) secretion into culture supernatant over time by human- and porcine-origin enterotoxigenic *E*. *coli* strains grown in CAYE-M medium. Strains were cultured at 37°C and 225 rpm in CAYE-M using a flask-to-medium ratio of 8.3:1. Samples of culture supernatants were obtained at 2, 4, 6 and 18 h of incubation, and LT concentrations in supernatant samples at each time interval were determined by GM1-ELISA. *LT concentrations in H10407 culture supernatant are significantly different (*P*<0.05) from that of all other strains at the corresponding time interval. ^†^LT concentrations in 3030–2 culture supernatant are significantly different (*P*<0.05) from that of all other porcine or 2534–86 derivative strains at the corresponding time interval. *eltAB*: strain is positive for LT genes by PCR. *estB*: strain is positive for STb gene by PCR. H = human-origin strain, P = porcine-origin strain, D = 2534–86 derivative strain.

**Fig 2:**
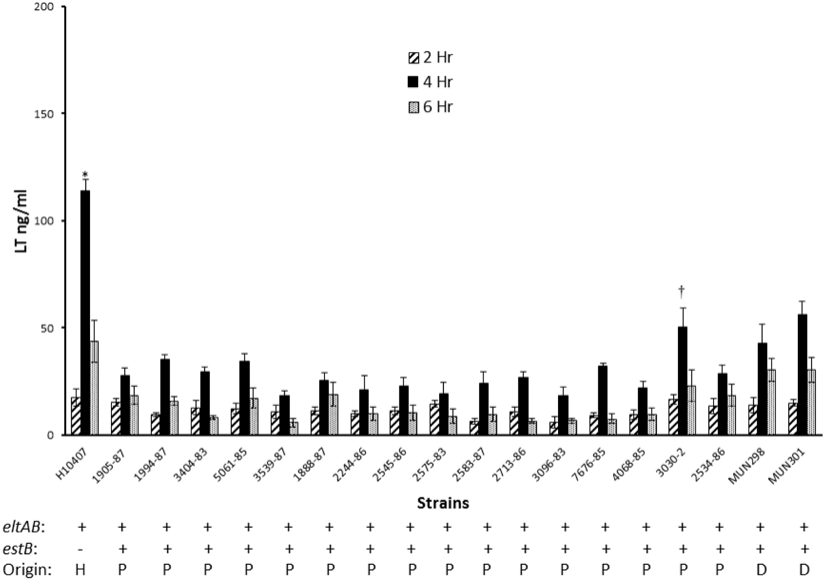
Heat-labile enterotoxin (LT) secretion into the periplasm over time by human- and porcine-origin enterotoxigenic *E*. *coli* strains grown in CAYE-M medium. Strains were cultured at 37°C and 225 rpm in CAYE-M using a flask-to-medium ratio of 8.3:1. Periplasmic extracts were prepared from samples of cell pellets obtained at 2, 4 and 6 h of culture, and LT concentrations in these extracts at each time interval were determined by GM1-ELISA. *LT concentrations in H10407 periplasmic extracts are significantly different (P<0.05) from that of all other strains at the corresponding time interval. ^†^LT concentrations in 3030–2 periplasmic extracts are significantly different (*P*<0.05) from that of all other porcine or 2534–86 derivative strains at the corresponding time interval. *eltAB*: strain is positive for the LT genes by PCR. *estB*: strain is positive for STb gene by PCR. H = human-origin strain, P = porcine-origin strain, D = 2534–86 derivative strain.

To confirm that other porcine-origin ETEC strains could secrete LT and determine whether LT secretion levels were detectably related to virulence, 16 ETEC strains isolated from pigs with severe disease (S1 Table) were compared with that of prototypic human-origin strain H10407. All strains were cultured in CAYE-M and sampled at 2, 4, and 6 h of culture to avoid significant contribution of LT to the supernatant by bacterial lysis. In addition, the supernatants at 18 h of culture were tested to determine whether any differences among strains remained so throughout the growth curve. LT was detected in the culture supernatants of all porcine WT strains at 2, 4 and 6 h, confirming the capacity to secrete the toxin (Fig. 1). At 2 h, the concentration of LT in culture supernatant for each of the strains did not differ significantly from that of H10407; however, at 4 and 6 h the concentrations were significantly lower than that of H10407 (*P*<0.05; Fig. 1). A notable finding was that by 6 h, strain 3030–2 had secreted a significantly higher concentration of LT into the supernatant compared to that of all other porcine WT strains tested (*P*<0.05) and this difference remained so through 18 h of culture (Fig. 1). Over all time points, all strains tested had a higher concentration of LT in the supernatant than in the periplasm (Fig. 2). As in the previous experiment, the highest concentration of LT in the periplasm was at 4 h (Fig. 2), and at this time the 3030–2 periplasmic LT concentration was significantly higher than that of the other WT porcine strains (*P*<0.05). To confirm that bacterial lysis was not a significant contributor to LT in the culture supernatant nor resulted in loss of LT from the periplasm during the exponential growth phase, each fraction was tested for alkaline phosphatase activity in the samples at 4 and 6 h of culture. No alkaline phosphatase activity was detected in the culture supernatants of any of the 16 WT porcine strains at either time interval, and all periplasmic extracts contained alkaline phosphatase activity, as expected. In contrast, at 12, 18 and 24 h of culture, alkaline phosphatase activity was present in both the culture supernatant and periplasmic fractions. These results confirmed that LT was present in the supernatants during the exponential phase by secretion and not by bacterial lysis, but during the stationary and death phases, a portion of the LT activity was present in the supernatants as a result of cell lysis.

### Virulence of Porcine-Origin ETEC Strains in Gnotobiotic Piglets

**Fig 3:**
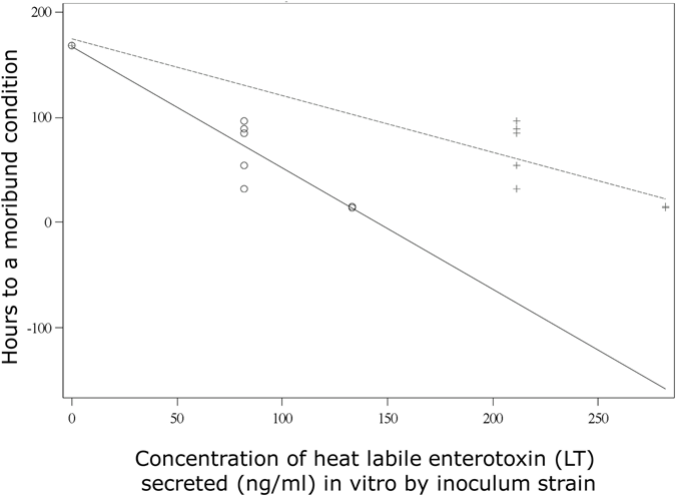
Linear regression between heat-labile enterotoxin (LT) secretion in culture and h to a moribund condition in gnotobiotic piglets inoculated with the corresponding *E*. *coli* strain. Concentration of LT in supernatants of 6-h cultures of strains G58–1 (LT^-^), 2534–86 (LT^+^), and 3030–2 (LT^+^; data shown in Fig. 1) and h-to-a-moribund-condition in piglets after inoculation with the same strains was used in the regression analysis.

Gnotobiotic piglets inoculated with strains 3030–2 or 2534–86 had an onset of diarrhea at 6 or 12 h PI, respectively, with subsequent passage of watery, clear-yellow fecal material. All piglets inoculated with strain 3030–2 or 2534–86 rapidly developed severe weight loss and dehydration, and became moribund; however, the clinical course with 3030–2 was more rapid. The mean time-to-a-moribund-condition with 3030–2 was 14.4 h PI, in contrast to 71.2 h PI for 2534–86 (*P*<0.001). Hence, among these 2 strains, 3030–2 was significantly more virulent. Non-enterotoxigenic strain G58–1 did not induce clinical illness, and piglets inoculated with this strain were euthanized at 96 h PI since their survival time would have been indefinite. A linear regression between LT secretion at 6 h of culture for these 3 strains and h-to-a-moribund-condition in inoculated gnotobiotic piglets was conducted. The model-adjusted coefficient of determination (R^2^) was 0.92 (*P* < 0.0001), indicating that approximately 92% of the variation in the time-to-a-moribund condition could be explained by the LT secretion level of the inoculum strain (Fig. 3). A second linear regression based on LT concentrations at 18 h of culture yielded a model-adjusted R^2^ of 0.8842 (*P* < 0.0001). This lower R^2^ value supported the hypothesis by Lasaro et al. [21] that virulence is more highly correlated with secretion than total production of LT, since a portion of the LT activity in the supernatants from 18 h cultures originated from lysed cells in the stationary-to-death phases.

**Fig 4:**
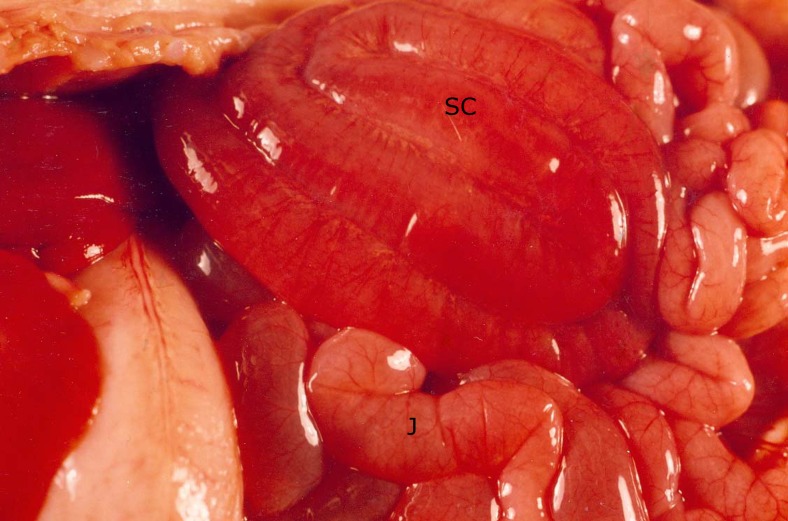
Photograph at necropsy of piglet 15 h after inoculation with enterotoxigenic *E*. *coli* strain 3030–2. Spiral colon (SC) is diffusely hemorrhagic, and jejunum (J) is hyperemic; both spiral colon and jejunum are distended with watery ingesta.

**Fig 5:**
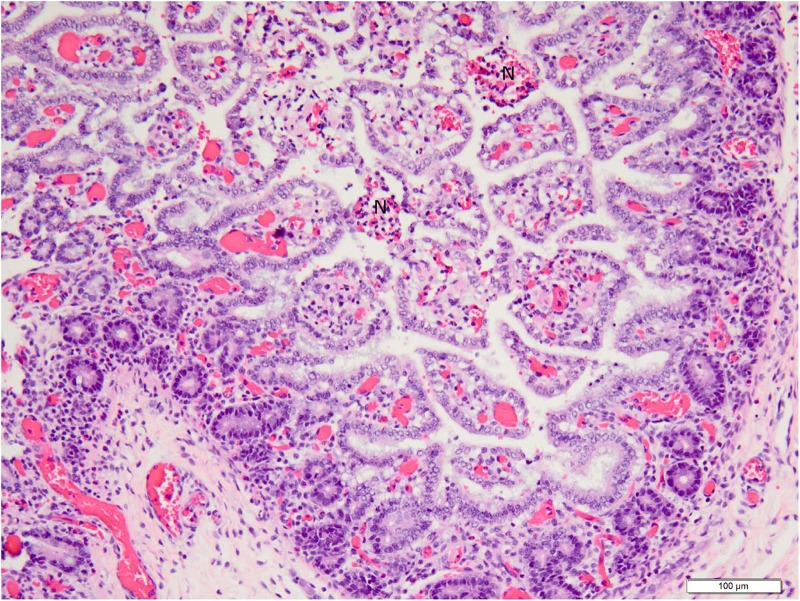
Low (20X objective) magnification photomicrograph of jejunum of piglet shown in Fig. 4. Mucosa and submucosa are diffusely hyperemic, and villi multifocally are necrotic. Necrotic villi (N) have hemorrhage in the lamina propria and loss of absorptive epithelium. Photomicrograph was taken of 4 μm-thick section of 10% neutral-buffered formalin-fixed, paraffin-embedded jejunal tissue stained with hematoxylin and eosin. Bar = 100 μm.

**Fig 6:**
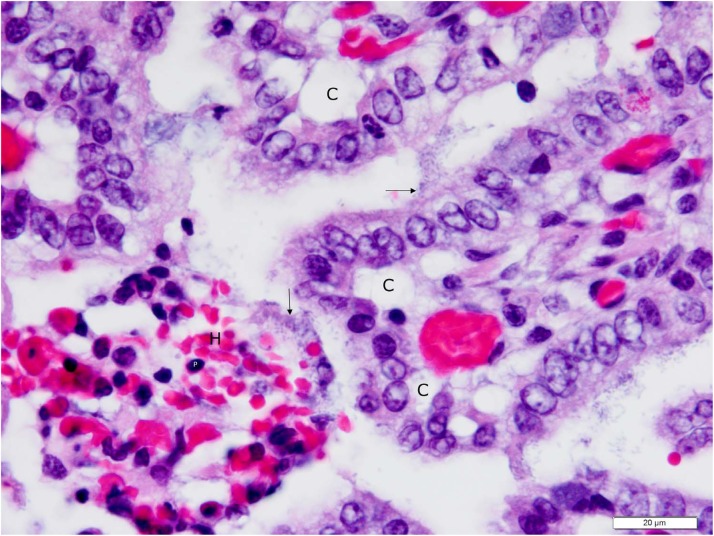
Higher (100X objective) magnification photomicrograph of jejunum shown in Fig. 5 with detail of necrotic and intact villi. The lamina propria of a necrotic villus is hemorrhagic (H), and contains numerous cells with pyknotic nuclei (P), indicating coagulation necrosis. The epithelium overlying this villus is absent, due to necrosis and sloughing of epithelial cells into the intestinal lumen. The exposed basal lamina of the necrotic villus is colonized with enterotoxigenic *E*. *coli* (vertical arrow), with bacteria having penetrated into the lamina propria with access to the microcirculation. The villi above and to the right of the necrotic villus have ETEC bacteria (horizontal arrow) colonizing the apical surfaces of absorptive epithelial cells. Many of these epithelial cells are in the process of sloughing as evidenced by the presence of clefts (C) between their basolateral surfaces and the underlying basal lamina. Photomicrograph was taken of 4 μm-thick section of 10% neutral-buffered formalin-fixed, paraffin-embedded jejunal tissue stained with hematoxylin and eosin. Bar = 20 μm.

**Fig 7:**
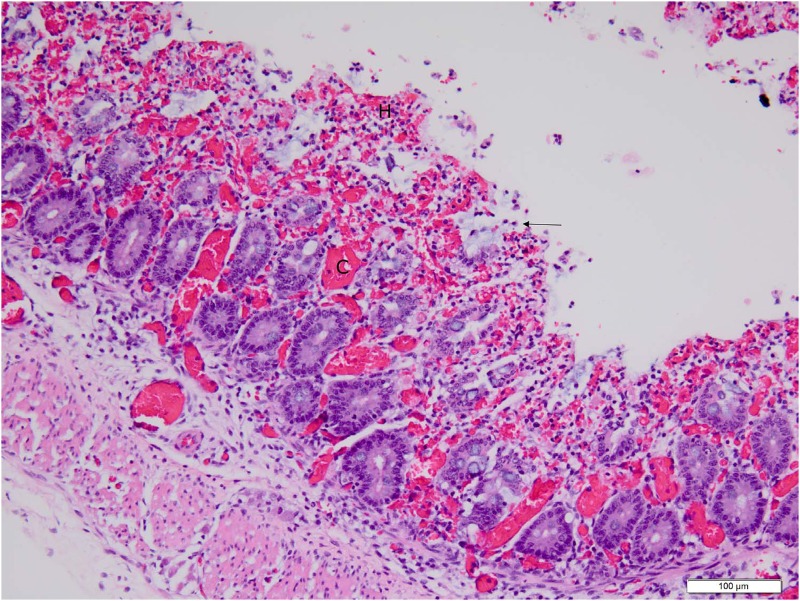
Low (20X objective) magnification photomicrograph of spiral colon of piglet shown in Fig. 4. Mucosa and submucosa are diffusely hyperemic and hemorrhagic; a markedly hyperemic venule (C) in the center of the field is seen. The epithelium on the mucosal surface is almost completely absent with the exception of a few cells (arrow) that are in the process of sloughing; architectural detail in the lamina propria and deep crypt epithelium are still intact at this point. Photomicrograph was taken of 4 μm-thick section of 10% neutral-buffered formalin-fixed, paraffin-embedded spiral colonic tissue stained with hematoxylin and eosin. Bar = 100 μm.

**Fig 8:**
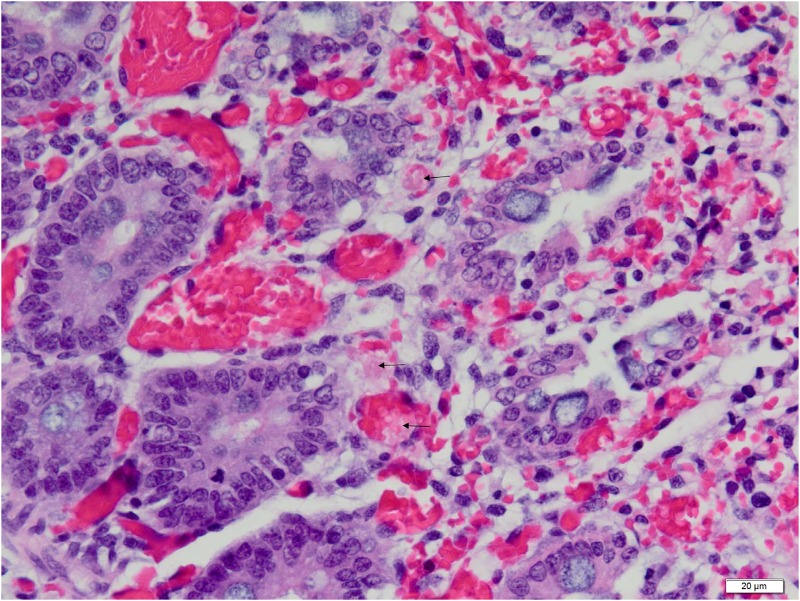
Higher (60X objective) magnification photomicrograph of the same field as that shown in Fig. 7. Eosinophilic platelet-fibrin thrombi (arrows) are seen in hyperemic capillaries and venules. Photomicrograph was taken of 4 μm-thick section of 10% neutral-buffered formalin-fixed, paraffin-embedded spiral colonic tissue stained with hematoxylin and eosin. Bar = 20 μm.

All piglets inoculated with 3030–2 or 2534–86 developed gross and microscopic lesions in the intestines compatible with shock (Figs. 4–8). Both small and large intestines were affected with hyperemia, hemorrhage and necrosis; these lesions were most severe in the mucosa, and were compatible with what we have previously described as hemorrhagic enteritis [4]. Histologically, necrotic epithelial cells in affected intestines had either sloughed or were in the process of it with formation of subepithelial clefts and exposure of the basal lamina. Intact small intestinal epithelium in all pigs inoculated with 2534–86 and 3030–2, but none inoculated with G58–1, had *E*. *coli* cells adherent to their apical surfaces, and in the case of pigs with lesions of shock had bacteria adherent to the exposed basal lamina. Platelet-fibrin thrombi and intravascular bacteria were seen in mucosal capillaries and venules of all piglets inoculated with 2534–86 and 3030–2, but in none of the piglets inoculated with G58–1. In all piglets inoculated with 2534–86 or 3030–2 but none inoculated with G58–1, either the respective inoculum strain, endotoxin activity or both were detected in blood samples obtained at necropsy. Mean endotoxin activity measured 0.49 ± 0.23 endotoxin units per ml in blood samples testing positive.

### Presence of Type II Secretion System in Porcine ETEC Strains

PCR analysis revealed the presence of both *gspD* and *gspK* genes in all porcine-origin WT strains shown in S1 Table with H10407 as a positive control (S4 Fig). Bioinformatic analyses of the genomic sequences of porcine LT^+^ WT strains 2534–86 and 3030–2, as well as that of porcine LT^-^ WT strain G58–1, further revealed that all 3 strains have a complete *gspC-M* operon with a high degree of similarity to that of H10407. A comparison of the *gspC-M* sequences revealed that 2534–86 shared 38.6–45.0% nucleotide and 94.3–99.8% amino acid identity with H10407, and 38.5–45.2% nucleotide and 27.3–76.2% amino acid identity with *V*. *cholerae* T2SS (Table 1). Similarly, 3030–2 shared 37.9–42.6% nucleotide and 20.0–76.2% amino acid identity with *V*. *cholerae* T2SS while having 37.8–44.7% nucleotide and 95.5–99.7% amino acid identity with the human ETEC strain H10407 (Table 1). Strain G58–1 shared 39.1–43.5% nucleotide and 15.7–61.2% amino acid identity with 2534–86, 37.3–65.1% nucleotide and 16.1–61.9% amino acid identity with H10407, 37.0–43.3% nucleotide and 16.7–64.6% amino acid identity with 3030–2, and 37.6–61.4% nucleotide and 16.3–62.6% amino acid identity with the *V*. *cholerae* T2SS (Table 2). Two other porcine ETEC strains in the NCBI database, UMNK88 and UMNF18, shared ≥95.7% nucleotide and ≥93.8% amino acid identity with 2534–86, whereas they collectively shared only 37.0–43.9% and 16.1–61.9% nucleotide and amino acid identity, respectively, with G58–1 (Table 3).

**Table 1:** Comparison of type II secretion system nucleotide and amino acid sequences of *Escherichia coli* strains 2534–86 and 3030–2 with that of *E*. *coli* strain H10407 and *Vibrio cholerae* strain TRH7000^a^.

**ETEC** ^**b**^ **/*Vibrio cholerae* gene**	***Vibrio cholerae* (TRH7000)**				***Escherichia coli* H10407**			
	**% Nucleotide Identity**		**% Amino acid Identity**		**% Nucleotide Identity**		**% Amino Acid Identity**	
	**2534–86**	**3030–2**	**2534–86**	**3030–2**	**2534–86**	**3030–2**	**2534–86**	**3030–2**
*gspC/espC*	42.4	39.3	31.7	29.5	45.0	44.7	99.3	98.2
*gspD/espD*	40.9	38.9	51.6	42.8	42.9	42.8	99.8	99.1
*gspE/espE*	44.2	37.9	66.4	69.3	44.6	37.8	99.4	99.7
*gspF/espF*	43.6	42.6	56.2	56.2	43.0	42.6	98.5	98.8
*gspG/espG*	43.0	41.6	76.2	76.2	40.4	40.6	98.0	98.7
*gspH/espH*	40.8	39.8	32.0	26.2	41.8	42.1	96.6	98.9
*gspI/espI*	40.7	40.3	42.4	39.0	43.2	42.2	94.3	96.7
*gspJ/espJ*	40.7	43.5	44.3	36.0	38.6	40.3	98.4	98.9
*gspK/espK*	45.2	42.5	39.6	39.6	43.2	42.7	98.5	99.7
*gspL/espL*	38.5	38.0	27.3	20.0	39.7	39.4	97.9	99.7
*gspM/espM*	43.9	42.4	29.0	28.3	42.0	41.4	94.9	95.5

^a^Accession numbers: 2534–86, AFDS01000066; 3030–2, AFDT01000052; H10407, AY056599; *Vibrio cholerae* TRH7000, L33796.

^b^ETEC: enterotoxigenic *Escherichia coli*.

**Table 2:** Comparison of type II secretion system nucleotide and amino acid sequences of *Escherichia coli* strain G58–1 with that of *E*. *coli* strains 2534–86, 3030–2, H10407, and *Vibrio cholerae* strain TRH7000^a^.

**ETEC** ^**b**^ **/ *Vibrio cholerae* gene**	***V*. *cholerae* TRH7000**		***E*. *coli* H10407**		***E*. *coli* 2534–86**		***E*. *coli* 3030–2**	
	**% NA ID** ^**c**^	**% AA ID** ^**c**^	**% NA ID**	**% AA ID**	**% NA ID**	**% AA ID**	**% NA ID**	**% AA ID**
*gspC/espC*	37.6	37.6	37.3	19.4	39.1	22.7	39.4	22.7
*gspD/espD*	53.9	42.8	50.2	44.5	42.5	43.9	41.4	42.8
*gspE/espE*	58.2	57.5	59.5	57.0	43.3	57.4	37.0	64.6
*gspF/espF*	41.4	43.6	42.4	45.1	42.9	44.7	42.7	44.8
*gspG/espG*	61.4	62.6	65.1	61.9	39.8	61.2	39.4	61.2
*gspH/espH*	43.4	26.1	40.7	19.8	43.5	20.7	42.3	20.3
*gspI/espI*	43.1	25.2	45.8	26.1	42.4	23.5	42.0	25.2
*gspJ/espJ*	44.2	22.7	43.2	21.8	42.8	21.3	43.3	21.8
*gspK/espK*	49.6	32.8	47.4	32.6	42.6	32.6	43.2	32.6
*gspL/espL*	43.4	18.3	41.1	19.9	40.6	15.7	39.9	18.4
*gspM/espM*	44.0	16.3	40.5	16.1	41.7	17.2	41.9	16.7

^a^Accession numbers: G58–1, AFDX01000001; 2534–86, AFDS01000066; 3030–2, AFDT01000052; H10407, AY056599; *Vibrio cholerae* TRH7000, L33796.

^b^ETEC: enterotoxigenic *Escherichia coli*.

^c^% NA ID = percent nucleotide identity; % AA ID = percent amino acid identity.

**Table 3:** Comparison of type II secretion system nucleotide and amino acid sequences of *Escherichia coli* strains G58–1, 2534–86, H10407, UMNK88, and UMNF18^a^.

**Gene**	**G58–1**				**H10407**				**2534–86**			
	**% Nucleotide Identity**		**% Amino Acid Identity**		**% Nucleotide Identity**		**% Amino Acid Identity**		**% Nucleotide Identity**		**% Amino Acid Identity**	
	**UMNK88**	**UMNF18**	**UMNK88**	**UMNF18**	**UMNK88**	**UMNF18**	**UMNK88**	**UMNF18**	**UMNK88**	**UMNF18**	**UMNK88**	**UMNF18**
*gspC*	38.7	39.9	22.1	22.2	44.7	44.0	97.1	95.3	97.5	96.1	97.8	95.3
*gspD*	43.0	43.9	43.9	43.9	43.3	42.2	99.7	99.7	98.6	97.2	99.9	99.9
*gspE*	43.6	43.7	57.2	57.2	43.9	44.7	99.2	99.2	98.6	98.2	99.4	99.4
*gspF*	41.9	42.6	44.8	45.1	42.1	41.5	98.8	99.5	97.6	97.3	99.3	98.5
*gspG*	39.9	39.0	61.9	61.9	40.5	40.5	98.7	99.3	97.6	98.2	98.0	98.7
*gspH*	43.3	43.7	21.2	20.7	40.7	40.4	95.5	94.9	98.7	98.9	98.9	98.3
*gspI*	37.0	41.2	25.2	26.1	42.2	43.9	95.1	99.2	97.3	96.0	95.9	95.1
*gspJ*	42.9	43.4	21.3	22.0	39.1	40.3	98.9	99.5	95.7	95.7	98.4	97.8
*gspK*	42.9	42.7	32.3	32.6	43.0	42.8	98.5	99.1	97.3	97.8	98.8	99.4
*gspL*	42.5	41.8	19.9	20.4	43.1	43.0	100.0	98.5	98.5	98.5	97.9	97.9
*gspM*	41.1	39.9	16.1	16.1	40.1	40.2	100.0	98.9	96.1	95.7	94.9	93.8

^a^Accession numbers: G58–1, AFDX01000001; 2534–86, AFDS01000066; 3030–2, AFDT01000052; H10407, AY056599; *Vibrio cholerae* TRH7000, L33796; UMNK88, CP002729; UMNF18, AGTD00000000.

**Fig 9:**
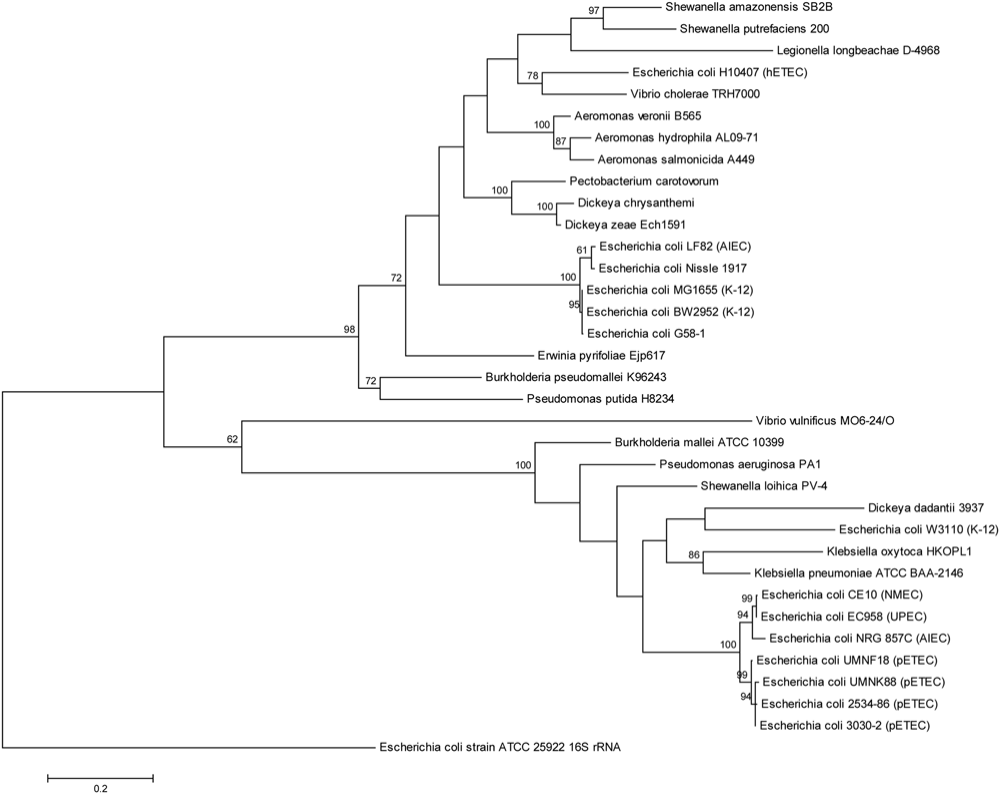
Maximum Likelihood phylogenetic tree generated by analyses of *gspE* and homolog sequences listed in S5 Table using MEGA6.

Maximum Likelihood phylogenetic trees were constructed using genetic sequences of *gspC* (S3 Table; S5 Fig), *gspD* (S4 Table; S6 Fig), *gspE* (S5 Table; Fig. 9) and corresponding homologs. In addition to *V*. *cholerae* TRH7000, porcine- and human-origin ETEC, and a subset of other bacterial strains that have been described in the literature to possess *gspC-M* homologs were used. All three trees generated showed that all four porcine ETEC strains (2534–86, 3030–2, UMNK88 and UMN18) clustered together in the same clade with high bootstrap values (≥ 60%). G58–1, however, clustered away from these ETEC strains. In trees generated by homologs of *gspD* and *gspE*, G58–1 clustered closer to H10407 and *V*. *cholerae* TRH7000.

Protein models were constructed for the 2534–86, 3030–2 and G58–1 predicted Gsp proteins based on structures of homologs in the Protein Data Bank (Table 4). The Qualitative Model Energy ANalysis (QMEAN)4 scores, which signify the quality of the model and its relatedness to the template used, showed that GspC, D, E, F, G, K and M have higher scores (i.e. closer to 1) while only GspL has a low QMEAN4 score and a very low negative Z-score value [1–3]. Similar results were obtained for protein models constructed with 3030–2 (Table 4). Both 2534–86 and 3030–2 had consistently higher QMEAN scores compared to those of G58–1. Furthermore, G58–1 protein modeling yielded structures with negative Z-scores in all instances, while both 2534–86 and 3030–2 had proteins that yielded positive or very low negative Z-scores which signified that the model elucidated is statistically better than the average models for those proteins.

**Table 4:** Protein modeling data for 2534–86, 3030–2 and G58–1 type II secretion system proteins GspC-M.

**Protein**	**Model Template** ^**a**^	**2534–86**		**3030–2**		**G58–1**	
		**QMEAN4 Score** ^**b**^	**Z-Score** ^**c**^	**QMEAN4 Score** ^**b**^	**Z-Score** ^**c**^	**QMEAN4 Score** ^**b**^	**Z-Score** ^**c**^
GspC	EpsC (2I4S)	0.971	1.37	0.971	1.37	0.657	-1.30
GspD	GspD (3EZJ)	0.895	0.85	0.801	0.12	0.729	-0.85
GspE	EpsE (1P9R)	0.754	-0.52	0.741	-0.63	0.685	-1.34
GspF	EpsF (2VMB)	0.662	-1.38	0.662	-1.38	0.647	-1.55
GspG	EpsG (3GN9)	0.894	0.85	0.899	0.90	0.671	-1.28
GspH	EpsH (2QV8)	0.579	-2.37	0.596	-2.18	0.688	-1.19
GspI	EpsI (2RET)	0.475	-2.34	0.491	-2.23	0.522	-1.92
GspJ	EpsJ (2RETE)	0.479	-3.61	0.502	-3.33	0.495	-3.28
GspK	EpsK (3CIO)	0.695	-1.24	0.699	-1.19	0.556	-3.31
GspL	EpsL (1W97)	0.214	-6.08	0.211	-6.11	0.429	-4.29
GspM	EpsM (1UV7)	0.750	-0.33	0.740	-0.40	0.608	-1.21

^a^Source of model template: EpsC, EpsE, EpsF, EpsH, EpsJ-M, *Vibrio cholerae* (strain not stated in literature); GspD, *E*. *coli* strain H10407; EpsG, EpsI, *V*. *vulnificus* (strain not stated in literature). Letters in parentheses are the Protein Data Bank (PDB) identification for the respective protein.

^b^QMEAN4 score (Range 0–1) is a composite score consisting of a linear combination of 4 statistical potential terms: (1) C-beta interaction energy, (2) all-atom pairwise energy, (3) solvation energy, and (4) torsion angle energy.

^c^Z-score: an estimate of the “degree of nativeness” of the structural features observed in a model by describing the likelihood that a model is of comparable quality to high-resolution experimental structures; it provides an estimate of the absolute quality of a model by relating it to reference structures solved by X-ray crystallography.

Proteins in T2SS that function as ATPases, in addition to the proton motive force, have been identified as the source of energy required for secretion [7,28]. Hence, we hypothesized that the ATP binding capacity of GspE for each of the strains tested would be related to LT secretion levels and virulence. To address this hypothesis, the protein model of 2534–86 GspE was aligned with that of H10407 GspE and *V*. *cholerae* TRH7000 EpsE using PyMol. While H10407 GspE and 2534–86 GspE both aligned with *V*. *cholerae* TRH7000 EpsE with a relative mean square (RMS) value of 0.083, H10407 and 2534–86 ATPases aligned with each other with a RMS of 0.001. PyRx was used to analyze the predicted ATP-binding capacities of the T2SS ATPases of each of the strains listed in the phylogenetic trees (Table 5). Of those strains which we had corresponding quantitative data for LT secretion, the predicted binding capacities, from highest to lowest, were H10407 (-8.8 kcal/mol), 3030–2 (-8.6 kcal/mol), 2534–86 (-8.5 kcal/mol), and G58–1 (-8.0 kcal/mol). MG1655, a K-12 *E*. *coli* strain, which was the origin of MUN302 used in this study, had a predicted binding capacity of-7.3 kcal/mol. For those strains for which we had virulence data (3030–2, 2534–86, and G58–1), a direct relationship was seen between virulence and the predicted ATP-binding capacity of the GspE.

**Table 5:** Predicted ATP-binding affinities of GspE homologs in different bacteria.

Strain	Predicted Binding Affinity (kcal/mol)
*Burkholderia mallei* ATCC 10399	-9.2
*Dickeya dadantii* 3937	-9.2
*Dickeya zeae* Ech1591	-9.2
*Pectobacterium carotovorum*	-9.2
*Shewanella putrefaciens* 200	-9.1
*Pseudomonas putida* H8234	-9.0
*Klebsiella pneumoniae* ATCC BAA-2146	-8.9
*Shewanella amazonensis* SB2B	-8.9
*Shewanella loihica* PV-4	-8.9
*Burkholderia pseudomallei* K96243	-8.8
*Dickeya chrysanthemi*	-8.8
*Escherichia coli* BW2952 (K-12)	-8.8
*Escherichia coli* H10407 (hETEC)	-8.8
*Escherichia coli* CE10 (NMEC)	-8.7
*Escherichia coli* NRG 857C (AIEC)	-8.7
*Escherichia coli* UMNF18 (pETEC)	-8.7
*Escherichia coli* W3110 (K-12)	-8.7
*Escherichia coli* 3030–2 (pETEC)	-8.6
*Pseudomonas aeruginosa* PA1	-8.6
*Escherichia coli* 2534–86 (pETEC)	-8.5
*Erwinia pyrifoliae* Ejp617	-8.2
*Escherichia coli* LF82 (AIEC)	-8.2
*Legionella longbeachae* D-4968	-8.1
*Vibrio vulnificus*	-8.1
*Aeromonas salmonicida* 449	-8.0
*Escherichia coli* G58–1	-8.0
*Escherichia coli* EC958 (UPEC)	-7.9
*Aeromonas hydrophila* AL09–71	-7.7
*Aeromonas veronii* B565	-7.6
*Escherichia coli* Nissle 1917	-7.4
*Escherichia coli* MG1655 (K-12)	-7.3
*Vibrio cholerae* TRH7000	-7.3
*Klebsiella oxytoca* HKOLP1	-6.7
*Escherichia coli* UMNK88 (pETEC)	-6.5

## Discussion

In the present study, we found that WT ETEC strains varied in LT secretion capacity, and this played a major role in determining virulence. One strain (3030–2) secreted significantly more LT than any other WT porcine strain tested, and also was significantly more virulent. When combining data for 3 strains that varied in virulence, we found that LT secretion, based on concentrations in supernatants from 6-h cultures explained 92% of the variation in time-to-a-moribund-condition. If the regression was run using LT concentrations in supernatants from 18-h cultures, the coefficient of determination was 89%. This lower R^2^ value supports the hypothesis by Lasaro et al. [21] that virulence is more highly correlated with secretion than total production of LT, since a portion of the LT activity in the supernatants from 18-h cultures originated from lysed cells. In a previous study, we found that approximately 58% of the variation in the rate of weight loss was explained by the LT production levels of the respective inoculum strain [9]. However, in that study, LT secretion per se was not measured, and the strains only included isogenic derivatives of 2534–86. The decision to euthanize gnotobiotic piglet experiments at 96 h PI if a moribund condition did not occur was somewhat arbitrary, but mainly based on animal welfare.

In previous studies, we determined that LT contributed more than STb to the severity of disease in 9-day-old gnotobiotic piglets inoculated with isogenic derivatives of strain 2534–86 [9]. In those studies, death phase (48- or 72-h) cultures treated with polymyxin B which caused further cell lysis were used to test for levels of LT production; hence, the capacity for the strain to secrete LT was not measured. In a more recent study, we found that these same strains secreted LT in 18-h cultures, but these studies did not test for attribution of LT secretion to virulence [11]. To our knowledge, as reported in the published literature, only 4 other porcine-origin strains had been tested for LT secretion, and the authors of these studies postulated that “the intact holotoxin transverses the outer membrane with newly synthesized LPS and becomes a component of the outer surface of *E*. *coli*” [20]. Besides the conclusion that LT is not fully secreted, this latter study provided no information about the virulence of the strains being tested, or the relationship, if any, between LT secretion and virulence.

In the present study, we detected LT in the supernatant by an optimized GM1-ELISA as early as 2 h of culture, and detected LT secretion by all 16 porcine WT ETEC strains tested, each isolated from cases of severe disease. Although 1 porcine strain was found to secrete relatively high levels of LT, we, similar to Gilligan and Robertson [15], also found that the levels of LT produced by porcine ETEC strains were lower than that of the prototypic human strain, H10407. Lasaro et al. [21], who analyzed 26 human ETEC isolates, reported that the levels of LT secreted by human strains can vary by as much as 50-fold. Although the culture media, conditions, and analytical methods used in our study differed in several respects, the levels of LT secreted by porcine ETEC strains in our study were within the general range of that of the human strains in the Lasaro et al. [21] study, with the exception of H10407. H10407 was originally isolated from a patient with severe, cholera-like diarrhea [12], and was found to secrete more LT than any of the 26 human test strains in the Lasaro et al. [21] study. In contrast to the conclusion by Gilligan and Robertson [15] that porcine ETEC strains produce less LT in complex medium than human strains, we conclude that strains from pigs and humans produce and secrete LT levels that are in general comparable to one another, whereas H10407 is more of an outlier.

We used a culture medium that had previously been shown to be optimal for LT secretion by H10407 and other human-origin strains [19,26] for detection of LT secretion by porcine ETEC strains. CAYE-M medium containing 0.25% glucose and adjusted to pH 8.5 has yielded the highest LT concentrations in culture supernatants in studies involving human-origin strains [19,26]. The molecular basis to explain the optimal nature of this medium for LT production and secretion has been apparent in recent studies. The cAMP repressor protein (CRP) is a repressor of *eltAB* transcription; glucose causes derepression of the *eltAB* promoter in H10407 by suppressing synthesis of cAMP, thereby decreasing cAMP availability to bind to the CRP and increasing transcription [5]. In contrast, CRP is a positive regulator of LT secretion and alkaline pH is a signal optimal for production and secretion of LT [16]. Based on the results reported herein, porcine ETEC strains would be expected to be affected by glucose and alkaline pH in the same manner as H10407; however, experiments specifically testing these hypotheses were not conducted.

Similar to the study of Tauschek et al. [34] with the prototypic human-origin strain H10407, we found that in porcine ETEC strains most of the LT secreted into the supernatant is not retained in the periplasm. The time at which the highest concentration of LT was detected in the periplasm by any strain tested was at 4 h PI. Furthermore, the LT levels in the supernatant did not increase significantly between 4 and 6 h, with some WT porcine strains showing similar or lower levels compared to the 4-h LT levels.

We found that all porcine-origin ETEC strains that had been genomically sequenced contained a T2SS with a high degree of amino acid identity to that of H10407, supporting the inference by Tauschek et al. [34] that the T2SS is highly conserved in ETEC. Interestingly, porcine-origin non-enterotoxigenic strain G58–1 was also found to contain the T2SS. This strain was originally isolated from a piglet with diarrhea, and is of a serotype (O101:K28) that is commonly enterotoxigenic, usually expressing K99 (F5) and/or 987P (F6) fimbria and STa [8,17,23]. Hence, this strain may have been an ETEC that lost one or more plasmids containing enterotoxin and fimbrial genes. The T2SS in G58–1 shares a similar level of nucleotide identity with both H10407 and 2534–86 but a much lower level of amino acid identity with the respective components of the secretion system. In addition, 3030–2, as well as the other 2 porcine ETEC strains in the NCBI database, all shared a very high degree of amino acid and nucleotide identity with each other, typically >93%, but a low level of nucleotide identity with the human ETEC strain H10407. This fact is emphasized by the phylogenetic trees generated using *gspC*, *gspD* and *gspE*, which showed that porcine ETEC have a similar clonal origin to each other while G58–1 clustered closer to H10407 and *V*. *cholerae* TRH7000. Since *gspC* and *gspD* encode substrate-specific components of the secretion pathway, this suggests a parallel evolution of the same secretion system, which is not surprising since all these organisms are subjected to the same evolutionary pressures that force them to evolve a functioning secretion system to take advantage of their enterotoxin [6]. It could be argued that both 2534–86 and 3030–2 developed a T2SS more efficient for recognizing the leader sequences of the enterotoxin subunits and their subsequent processing in the periplasm. This may have happened after the acquisition of the LT containing plasmid resulting in a secretion system that shares a high protein identity with H10407, while G58–1 which shares a closer clonal match to H10407, has a low protein identity with the H10407 T2SS.

GspE (EpsE) binds and hydrolyzes ATP, thereby providing energy for pseudopilus assembly and protein secretion [7,28]. The results of protein modeling of these ATPases showed that different T2SS have different predicted binding capacities, which might in part explain the different secretion capabilities of different strains of both animal and human ETEC. However, further experiments are needed to test this hypothesis. The predicted binding affinities were generated by using the respective GspE monomer of the T2SS. However, it has been shown that the hexameric form of GspE has a much higher ATPase activity than the monomeric one, and is thought to be the functional form in nature [7,28]. Therefore, it can be assumed that the ATPase activities of GspE in the strains tested herein might be different in nature from their predicted values based on protein models.

## Supporting Information

S1 Fig Heat-labile enterotoxin (LT) concentrations in supernatants of cultures of human- and porcine-origin enterotoxigenic *E*. *coli*. Strains were cultured at 37°C and 225 rpm in Casamino Acids yeast extract medium-Mundell (CAYE-M) medium containing 0.25% glucose, pH 8.5 using a flask-to-medium ratio of 8.3:1. Samples of culture supernatant were obtained at 4, 8, 12, and 24 h of incubation, and LT concentrations in these samples were measured by GM1-ELISA. A human-origin strain is represented by H10407, whereas porcine-origin strains are represented by wild type 2534–86 and derivative strains, MUN298 (LT^+^, Δ*estB*, pBR322::*estB*) and MUN299 (LT^-^ Δ*eltAB*).
(TIF)

S2 Fig Heat-labile enterotoxin (LT) concentrations in periplasmic extracts of human- and porcine-origin enterotoxigenic *E*. *coli* strains. Strains were cultured at 37°C and 225 rpm in Casamino Acids yeast extract medium-Mundell (CAYE-M) medium containing 0.25% glucose, pH 8.5 using a flask-to-medium ratio of 8.3:1. Periplasmic extracts were prepared from cell pellets of samples obtained at 4, 8, 12, and 24 h of culture, and LT concentrations were measured by GM1-ELISA. A human-origin strain is represented by H10407, whereas porcine-origin strains are represented by wild type 2534–86 and derivative strains, MUN298 (LT^+^, Δ*estB*, pBR322::*estB*) and MUN299 (LT^-^ Δ*eltAB*).
(TIF)

S3 Fig Growth curves of human- and porcine-origin enterotoxigenic *E*. *coli* strains. Strains were cultured at 37°C and 225 rpm in Casamino Acids yeast extract medium-Mundell (CAYE-M) medium containing 0.25% glucose, pH 8.5 using a flask-to-medium ratio of 8.3:1. Samples were obtained at 0, 4, 8, 12 and 24 h of culture and from these samples the OD_600_, colony-forming units (CFU)/ml and pH values were determined. The CFU/ml were determined by serial 10-fold dilution and plating on LB agar. A human-origin strain is represented by H10407, whereas porcine-origin strains are represented by wild type 2534–86 and derivative strains, MUN298 (LT^+^, Δ*estB*, pBR322::*estB*) and MUN299 (LT^-^ Δ*eltAB*).
(TIF)

S4 Fig Detection of *gspD* and *gspK* in wild type porcine-origin enterotoxigenic *E*. *coli* strains (ETEC) by polymerase chain reaction (PCR) assay. PCR assays to determine the existence of the T2SS in porcine ETEC stains were conducted using primers gspDF (5-TTCGGAAATCGCCCGCGTGC) and gspDR (5-TCCACCTTCGAGACTTCC) to generate a 1.0-kb fragment of *gspD*, and primers gspKF (5-GCAGCAGGTGACTAACGGC) and gspKR (5-CAGGGCTTAACCACGGGTC) to generate a 1.2-kb fragment of *gspK* [34]. PCR reactions were conducted using a 95°C initial denaturation for 1 min, followed by 30 cycles of 95°C (30 sec), 60°C (30 sec), and 68°C (90 sec), and a final extension at 72°C for 10 min. Electrophoresis was performed using a 2% agarose- tris acetate ethanol (TAE) gel, supplemented with 0.5 µg/ ml of ethidium bromide. Human-origin strain H10407 was used as a positive control for the presence of *gspD* and *gspK*, and a lane lacking DNA was used as negative control. Amplicons of the appropriate sizes for *gspD* and *gspK* were seen in the lanes containing DNA from H10407 and all porcine-origin ETEC strains (arrows), but not in the negative control lane.
(TIF)

S5 Fig Maximum Likelihood phylogenetic tree generated by analyses of *gspC* and homolog sequences listed in S3 Table using MEGA6. (TIF)

S6 Fig Maximum Likelihood phylogenetic tree generated by analyses of *gspD* and homolog sequences listed in S4 Table using MEGA6. (TIF)

S1 Table *Escherichia coli* strains used in this study. (DOCX)

S2 Table Y1 adrenal cell assay results. (DOCX)

S3 Table Genetic sequences of *gspC* homologs used for generating the Maximum Likelihood phylogenetic tree. (DOCX)

S4 Table Genetic sequences of *gspD* homologs used for generating the Maximum Likelihood phylogenetic tree. (DOCX)

S5 Table Genetic sequences of *gspE* homologs used for generating the Maximum Likelihood phylogenetic tree. (DOCX)
