# Supplementary figures and images for: Relationship between Heat-Labile Enterotoxin Secretion Capacity and Virulence in Wild Type Porcine-Origin Enterotoxigenic Escherichia coli Strains
Source: PLoS One. 2015 Mar 13;10(3):e0117663. doi: 10.1371/journal.pone.0117663 (PMC4358887; doi:10.1371/journal.pone.0117663)

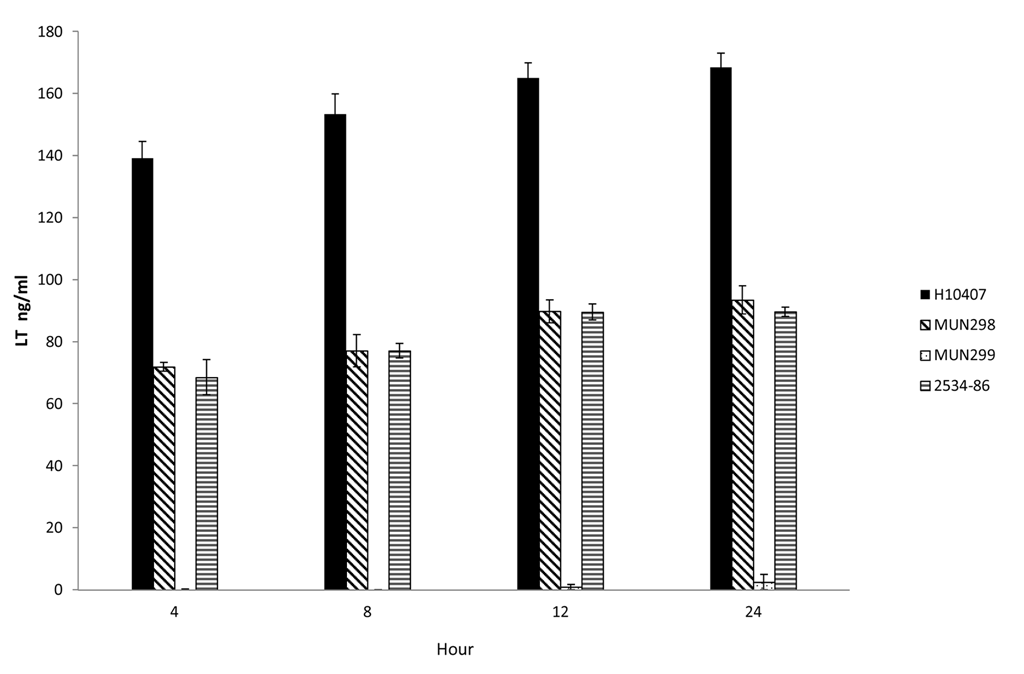

Supplement: S1 Fig — Strains were cultured at 37°C and 225 rpm in Casamino Acids yeast extract medium-Mundell (CAYE-M) medium containing 0.25% glucose, pH 8.5 using a flask-to-medium ratio of 8.3:1. Samples of culture supernatant were obtained at 4, 8, 12, and 24 h of incubation, and LT concentrations in these samples were measured by GM1-ELISA. A human-origin strain is represented by H10407, whereas porcine-origin strains are represented by wild type 2534–86 and derivative strains, MUN298 (LT+, ΔestB, pBR322::estB) and MUN299 (LT- ΔeltAB). (TIF) [file pone.0117663.s001.tif]

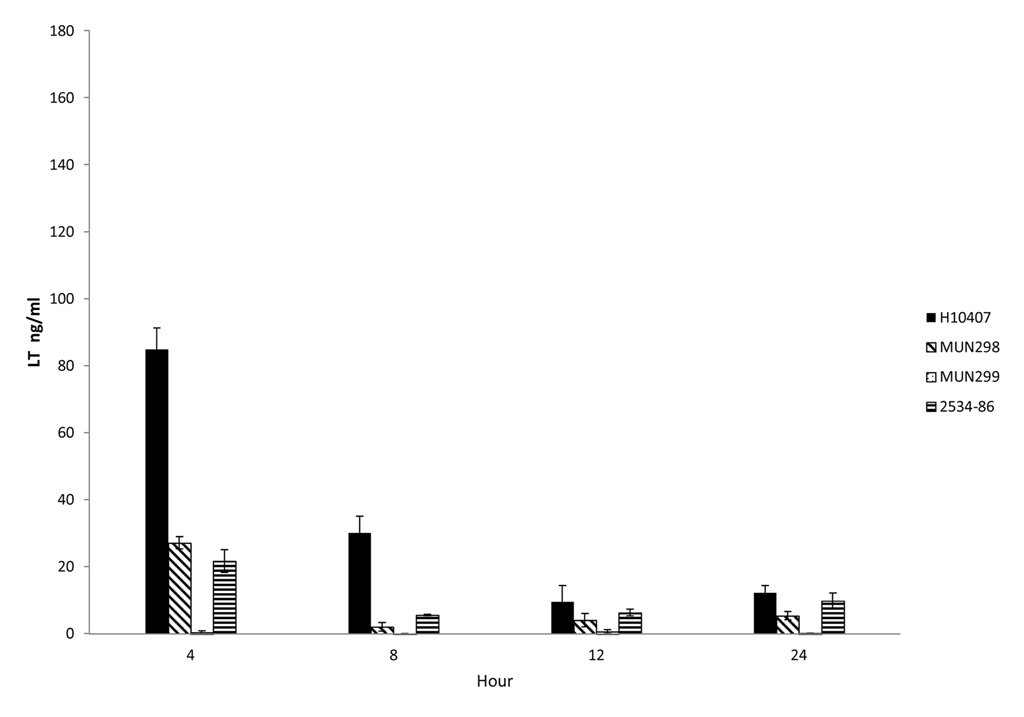

Supplement: S2 Fig — Strains were cultured at 37°C and 225 rpm in Casamino Acids yeast extract medium-Mundell (CAYE-M) medium containing 0.25% glucose, pH 8.5 using a flask-to-medium ratio of 8.3:1. Periplasmic extracts were prepared from cell pellets of samples obtained at 4, 8, 12, and 24 h of culture, and LT concentrations were measured by GM1-ELISA. A human-origin strain is represented by H10407, whereas porcine-origin strains are represented by wild type 2534–86 and derivative strains, MUN298 (LT+, ΔestB, pBR322::estB) and MUN299 (LT- ΔeltAB). (TIF) [file pone.0117663.s002.tif]

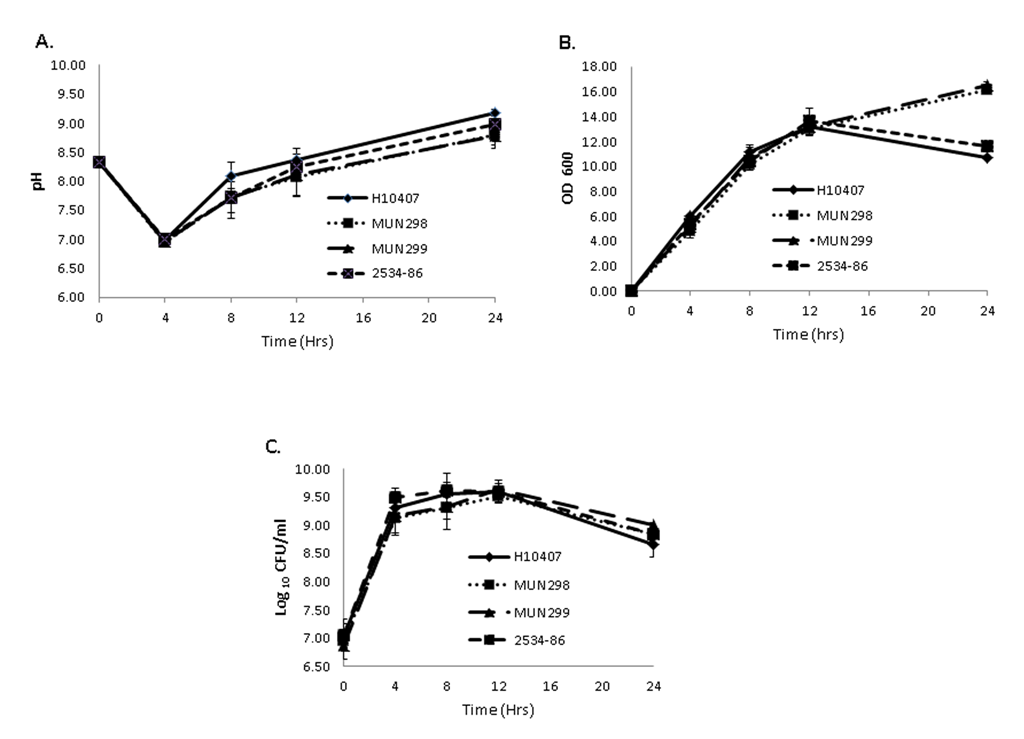

Supplement: S3 Fig — Strains were cultured at 37°C and 225 rpm in Casamino Acids yeast extract medium-Mundell (CAYE-M) medium containing 0.25% glucose, pH 8.5 using a flask-to-medium ratio of 8.3:1. Samples were obtained at 0, 4, 8, 12 and 24 h of culture and from these samples the OD600, colony-forming units (CFU)/ml and pH values were determined. The CFU/ml were determined by serial 10-fold dilution and plating on LB agar. A human-origin strain is represented by H10407, whereas porcine-origin strains are represented by wild type 2534–86 and derivative strains, MUN298 (LT+, ΔestB, pBR322::estB) and MUN299 (LT- ΔeltAB). (TIF) [file pone.0117663.s003.tif]

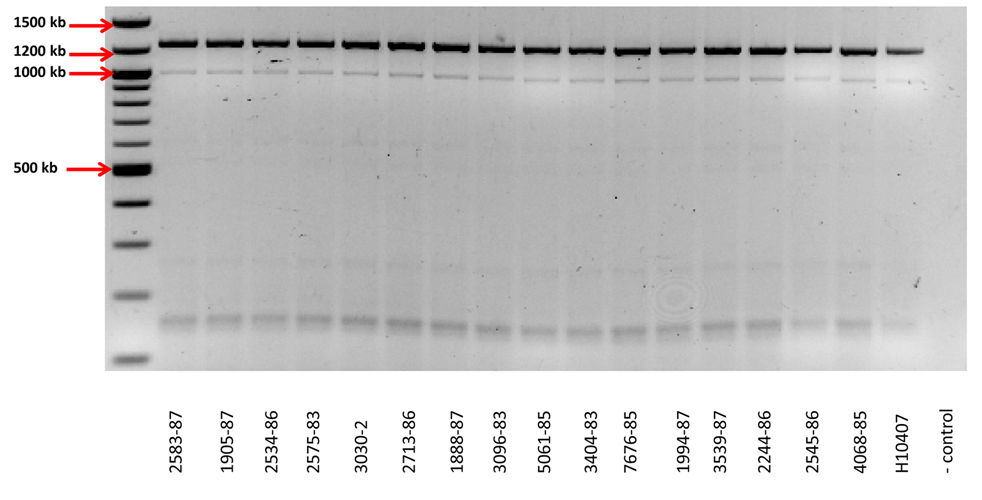

Supplement: S4 Fig — PCR assays to determine the existence of the T2SS in porcine ETEC stains were conducted using primers gspDF (5-TTCGGAAATCGCCCGCGTGC) and gspDR (5-TCCACCTTCGAGACTTCC) to generate a 1.0-kb fragment of gspD, and primers gspKF (5-GCAGCAGGTGACTAACGGC) and gspKR (5-CAGGGCTTAACCACGGGTC) to generate a 1.2-kb fragment of gspK [34]. PCR reactions were conducted using a 95°C initial denaturation for 1 min, followed by 30 cycles of 95°C (30 sec), 60°C (30 sec), and 68°C (90 sec), and a final extension at 72°C for 10 min. Electrophoresis was performed using a 2% agarose- tris acetate ethanol (TAE) gel, supplemented with 0.5 µg/ ml of ethidium bromide. Human-origin strain H10407 was used as a positive control for the presence of gspD and gspK, and a lane lacking DNA was used as negative control. Amplicons of the appropriate sizes for gspD and gspK were seen in the lanes containing DNA from H10407 and all porcine-origin ETEC strains (arrows), but not in the negative control lane. (TIF) [file pone.0117663.s004.tif]

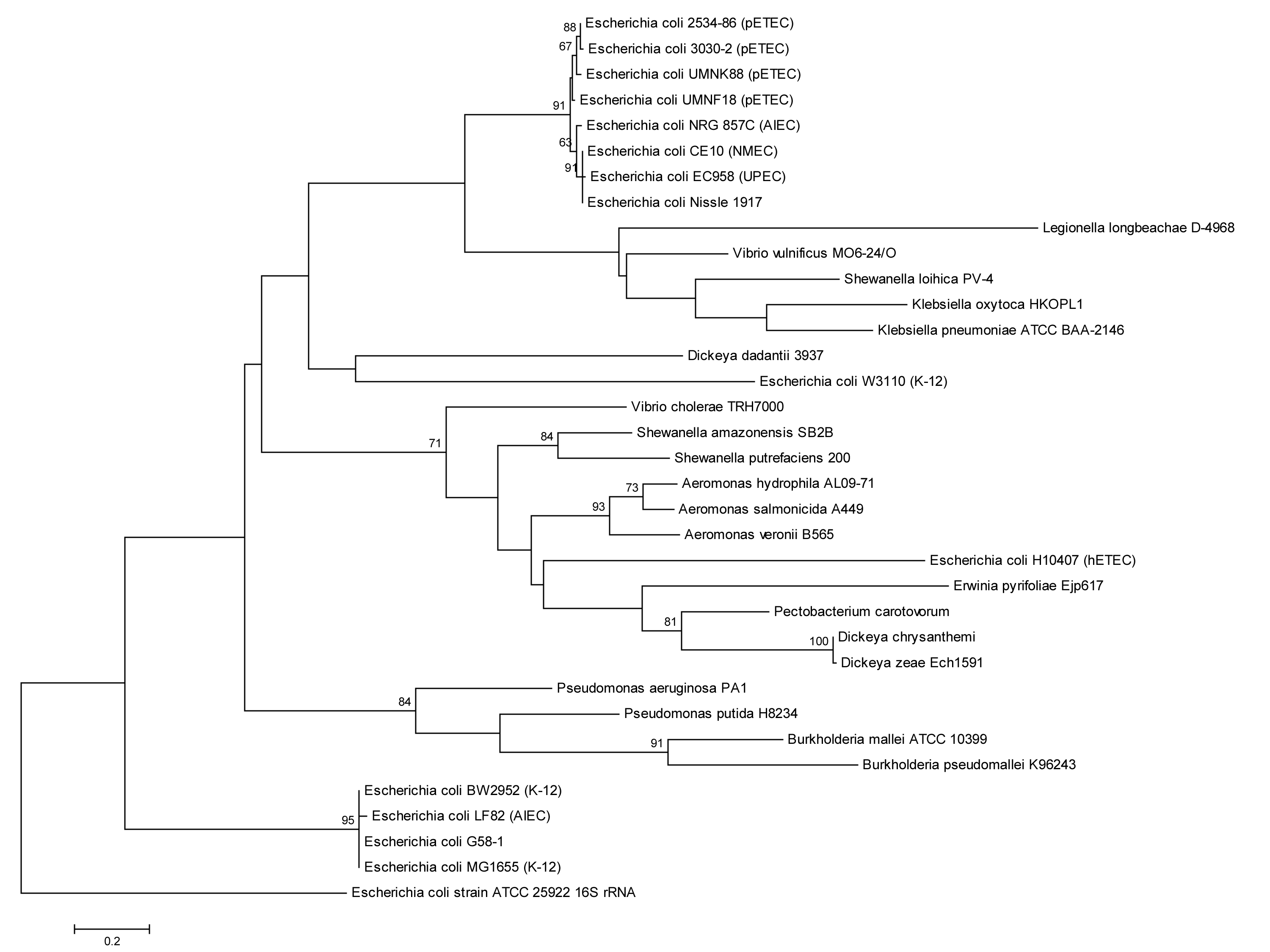

Supplement: S5 Fig — (TIF) [file pone.0117663.s005.tif]

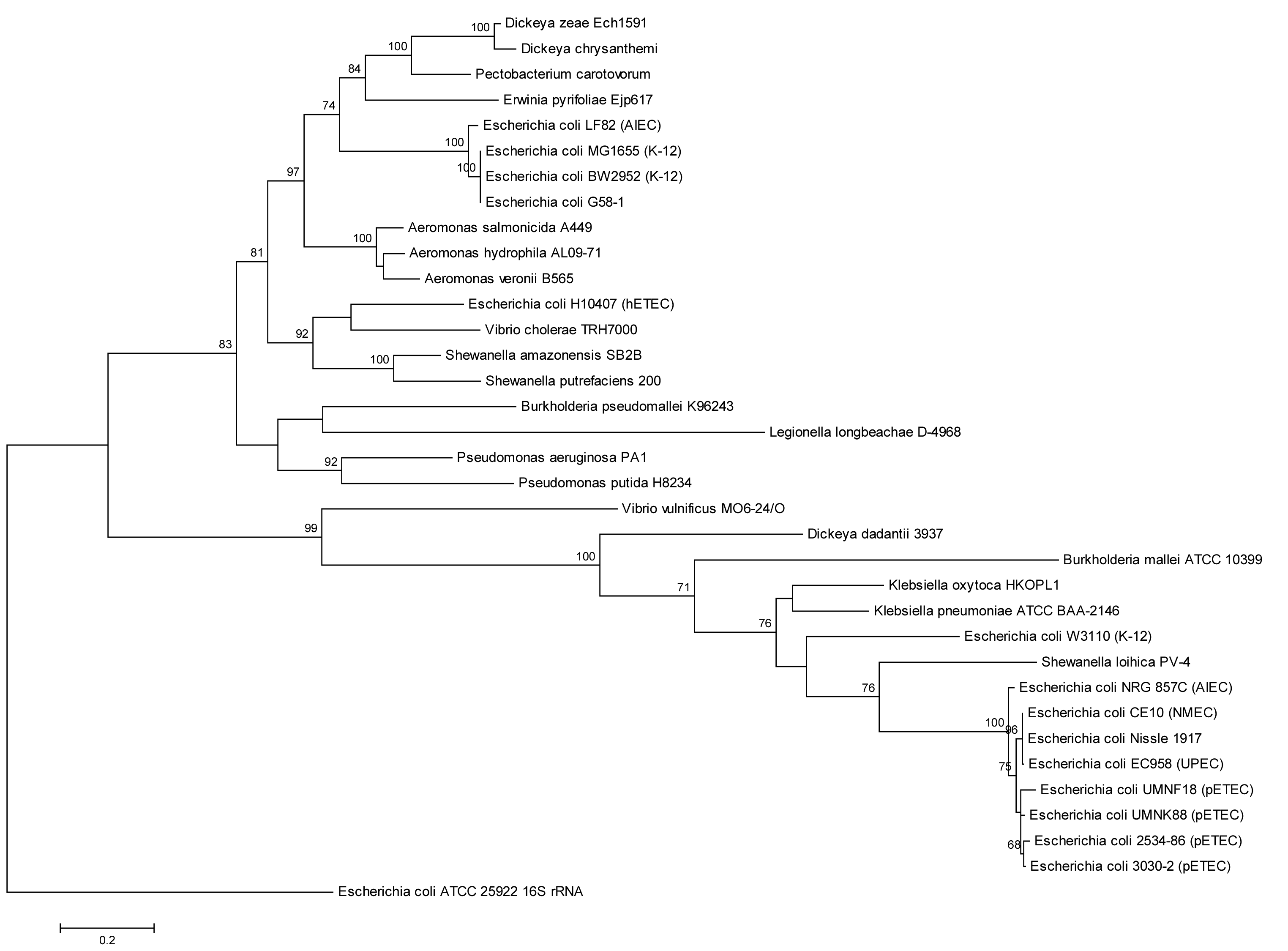

Supplement: S6 Fig — (TIF) [file pone.0117663.s006.tif]
