# Supplementary material for: Relationship between Heat-Labile Enterotoxin Secretion Capacity and Virulence in Wild Type Porcine-Origin Enterotoxigenic Escherichia coli Strains
Source: PLoS One. 2015 Mar 13;10(3):e0117663. doi: 10.1371/journal.pone.0117663 (PMC4358887; doi:10.1371/journal.pone.0117663)
Supplement: S1 Table — (DOCX) [file pone.0117663.s007.docx]

**Table S1.** *Escherichia coli* strains used in this study.

| **Strain** | **Description^a^** | **Reference** |
| --- | --- | --- |
| H10407 | O78:K80:H11, CFA/1^+^, LT^+^, STp^+^, STh^+^, HWT (adult, cholera-like D) | [12] |
| 2534-86 | O8:K87:H^-^, F4ac^+^, LT^+^, STb^+^, PWT (2 wks, D, HE) | [25] |
| WAM2317 | O8:K87:H^-^, F4ac^+^, LT^+^, STb^+^, spontaneous Nal^r^ mutant of 2534-86 | [25] |
| MUN297 | O8:K87:H^-^, F4ac^+^, LT^+^, STb^-^, Nal^r^, Km^r^, Δ*estB*::Km^r^ derivative of WAM2317 | [9] |
| MUN298 | O8:K87:H^-^, F4ac^+^, LT^+^, STb^+^, Nal^r^, Km^r^, Amp^r^, MUN297/pBR322::*estB* | [9] |
| MUN299 | O8:K87:H^-^, F4ac^+^, LT^-^, STb^+^, Nal^r^, Km^r^, Δ*eltAB*::Km^r^ derivative of WAM2317 | [9] |
| MUN300 | O8:K87:H^-^, F4ac^+^, LT^-^, STb^-^, Nal^r^, Km^r^, Cm^r^, Δ*estB*::Cm^r^ derivative of MUN299 | [9] |
| MUN301 | O8:K87:H^-^, F4ac^+^, LT^+^, STb^-^, Nal^r^, Km^r^, Cm^r^, Amp^r^, MUN300/pBR322::*eltAB*) | [9] |
| MUN302 | LT^+^, STb^-^, DH5α/pBR322::*eltAB* | [9] |
| MUN303 | LT^-^, STb^-^, DH5α/pBR322 | [9] |
| DH5α | K-12 laboratory strain, F^-^ Φ80*lac*ZΔM15 Δ(*lacZYA*-*argF*) U169 *recA1* *endA1* *hsdR17* (rK^-^, mK^+^) *phoA* *supE44* λ^-^ *thi*-1 *gyrA96* *relA1* | [18] |
| 1836-2 | O8:H4:F4ac, LT^-^, STb^-^, PWT (piglet of unknown age, ND) | [36] |
| G58-1 | O101:K28:H^-^:F^-^, LT^-^, STb^-^, PWT (piglet of unknown age, D) | [8] |
| 1905-87 | O8:H19:F4, LT^+^, STb^+^, PWT (3 wks, D) | [9] |
| 1994-87 | O8:H19:F4, LT^+^, STb^+^, PWT (4 wks, D, SD, HE) | [9] |
| 3404-83 | O149:H19:F4, LT^+^, STb^+^ PWT (8 wks, D, HE) | [9] |
| 5061-85 | O149:H^-^:F4, LT^+^, STb^+^, PWT (3-5 d, D, HE) | [9] |
| 3539-87 | O157:H^-^:F4, LT^+^, STb^+^, PWT (1-3 d, D) | [9] |
| 1888-87 | O149:H19:F4, LT^+^, STb^+^, PWT (8 wks, D, HE) | [9] |
| 2244-86 | O149:H19:F4, LT^+^, STb^+^, PWT (4 wks, SD, HE) | [9] |
| 2545-86 | O149:H19:F4, LT^+^, STb^+^, PWT (5-6 wks, D, HE) | [9] |
| 2575-83 | O149:H^-^:F4, LT^+^, STb^+^, PWT (2-3 wks, D, SD, HE) | [9] |
| 2583-87 | O157:H^-^:F4, LT^+^, STb^+^, PWT (5 wks, D, SD, HE) | [9] |
| 2713-86 | O149:H19:F4, LT^+^, STb^+^, PWT (2 d, D, HE) | [9] |
| 3096-83 | O149:H19:F4, LT^+^, STb^+^, PWT (3 wks, SD, HE) | [9] |
| 7676-85 | O149:H^-^:F4, LT^+^, STb^+^, PWT (4 wks, SD, HE) | [9] |
| 4068-85 | O149:H^-^:F4, LT^+^, STb^+^, PWT (4 wks, SD, HE) | [9] |
| 3030-2 | O157:H^-^:F4ac, LT^+^, STb^+^, PWT (weanling, D, HE) | [14] |

^a^O antigen: K antigen (if known): H antigen, F (fimbrial) antigen (subtype included if known); CFA, colonization factor antigen; LT, heat-labile enterotoxin; STp, heat-stable enterotoxin-a, porcine; STh, heat-stable enterotoxin-a, human; STb, heat-stable enterotoxin-b; HWT, human-derived wild type; PWT, porcine-derived wild type; wks or d, age of pig in weeks or days from which strain was isolated; D, diarrhea; ND, no diarrhea; SD, sudden death; HE, hemorrhagic enteritis was detected grossly and/or histologically in the piglet from which the strain was originally isolated or experimentally in gnotobiotic piglets subsequently inoculated with the strain.; Nal^r^, nalidixic acid-resistant; Km^r^, kanamycin-resistant; Cm^r^, chloramphenicol-resistant; Amp^r^, ampicillin-resistant; *eltAB*, genes encoding LT; *estB*, gene encoding STb.
