# Supplementary material for: Relationship between Heat-Labile Enterotoxin Secretion Capacity and Virulence in Wild Type Porcine-Origin Enterotoxigenic Escherichia coli Strains
Source: PLoS One. 2015 Mar 13;10(3):e0117663. doi: 10.1371/journal.pone.0117663 (PMC4358887; doi:10.1371/journal.pone.0117663)
Supplement: S2 Table — (DOCX) [file pone.0117663.s008.docx]

**Table S2.** Y1 adrenal cell assay results.

| **Strain** | **GM1 ELISA activity** | **Titer in Y1 cells*** | | |
| --- | --- | --- | --- | --- |
|  |  | **After 4 hours** | **After 24 hours** |  |
| MUN297 | + | - | 1:40 |  |
| MUN299 | - | - |  |  |
| MUN300 | - | - |  |  |
| MUN301 | + | - | 1:20 |  |
| MUN302 | + | - | 1:20 |  |
| H10407 | + | - | 1:40 |  |
| 1836-2 | - | - |  |  |
| G58-1 | - | - |  |  |
| DH5α | - | - |  |  |
| 2534-86 | + | - | 1:20 |  |
| WAM2317 | + | - | 1:20 |  |
| 3030-2 | + | - | 1:20 |  |

*Titer reflects the highest dilution at which cell rounding involving >50% of the cells was detected.
