# Supplementary material for: Relationship between Heat-Labile Enterotoxin Secretion Capacity and Virulence in Wild Type Porcine-Origin Enterotoxigenic Escherichia coli Strains
Source: PLoS One. 2015 Mar 13;10(3):e0117663. doi: 10.1371/journal.pone.0117663 (PMC4358887; doi:10.1371/journal.pone.0117663)
Supplement: S3 Table — (DOCX) [file pone.0117663.s009.docx]

**Table S3.** Genetic sequences of *gspC* homologs used for generating the Maximum Likelihood phylogenetic tree.^a^

| **Strain** | **GenBank Accession No.** |
| --- | --- |
| *Aeromonas hydrophila* AL09-71 | CP007566.1\|:638829-639674 |
| *Aeromonas salmonicida* 449 | CP000644.1\|:4067315-4068187 |
| *Aeromonas veronii* B565 | CP002607.1\|:3257300-3258139 |
| *Burkholderia mallei* ATCC 10399 | CH899680.1\|:193836-194246 |
| *Burkholderia pseudomallei* K96243 | BX571965.1\|: 13452-13862 |
| *Dickeya chrysanthemi* | L02214.1\|ERWOUTCM:730-1548 |
| *Dickeya dadantii* 3937 | CP002038.1\|:2888814-2889302 |
| *Dickeya zeae* Ech1591 | CP001655.1\|:1473340-1474158 |
| *Erwinia pyrifoliae* Ejp617 | CP002124.1\|:358437-358898 |
| *Escherichia coli* 2534-86 (pETEC) | AFDS01000066.1\|:14963-15793 |
| *Escherichia coli* 3030-2 (pETEC) | AFDT01000052.1\|:16960-17790 |
| *Escherichia coli* BW2952 (K-12) | CP001396.1\|:3340757-3341572 |
| *Escherichia coli* CE10 (NMEC) | CP001396.1\|:3340757-3341572 |
| *Escherichia coli* EC958 (UPEC) | HG941718.1\|:3389867-3390697 |
| *Escherichia coli* G58-1 | AFDX01000036.1\|:16094-16807 |
| *Escherichia coli* H10407 (hETEC) | AY056599.1\|:2234-3064 |
| *Escherichia coli* LF82 (AIEC) | CU651637.1\|:3498687-3499502 |
| *Escherichia coli* MG1655 (K-12) | U00096.3\|:3455578-3456393 |
| *Escherichia coli* Nissle 1917 | CP007799.1\|:3440567-3441526 |
| *Escherichia coli* NRG 857C (AIEC) | CP001855.1\|:3112931-3113890 |
| *Escherichia coli* UMNF18 (pETEC) | AGTD01000001.1\|:3643451-3644281 |
| *Escherichia coli* UMNK88 (pETEC) | CP002729.1\|:3592465-3593295 |
| *Escherichia coli* W3110 (K-12) | AP009048.1\|:4184023-4184838 |
| *Klebsiella oxytoca* HKOLP1 | CP004887.1\|:5024742-5025233 |
| *Klebsiella pneumoniae* ATCC BAA-2146 | CP006659.1\|:957129-957971 |
| *Legionella longbeachae* D-4968 | ACZG01000001.1\|:936613-937122 |
| *Pectobacterium carotovorum* | X70049.1\|:74-931 |
| *Pseudomonas aeruginosa* PA1 | CP004054.1\|:1075982-1076638 |
| *Pseudomonas putida* H8234 | CP005976.1\|:1106032-1106457 |
| *Shewanella amazonensis* SB2B | CP000507.1\|:197297-198220 |
| *Shewanella loihica* PV-4 | CP000606.1\|:4317974-4318891 |
| *Shewanella putrefaciens* 200 | CP002457.1\|:425346-426272 |
| *Vibrio cholerae* TRH7000 | L33796.1\|VIBEPSCN:213-1130 |
| *Vibrio vulnificus* | CP002469.1\|:3073737-3074666 |
| *Escherichia coli strain* ATCC 25922 16S rRNA | DQ360844.1:86278349 |

^a^pETEC: porcine-derived enterotoxigenic *Escherichia coli*; NMEC: neonatal meningitis *E. coli*; UPEC: uropathogenic *E. coli*; hETEC: human-derived enterotoxigenic *E. coli*; AIEC: adherent-invasive *E. coli*.
