# Supplementary material for: Relationship between Heat-Labile Enterotoxin Secretion Capacity and Virulence in Wild Type Porcine-Origin Enterotoxigenic Escherichia coli Strains
Source: PLoS One. 2015 Mar 13;10(3):e0117663. doi: 10.1371/journal.pone.0117663 (PMC4358887; doi:10.1371/journal.pone.0117663)
Supplement: S4 Table — (DOCX) [file pone.0117663.s010.docx]

**Table S4.** Genetic sequences of *gspD* homologs used for generating the Maximum Likelihood phylogenetic tree.^a^

| **Strain** | **GenBank Accession No.** |
| --- | --- |
| *Aeromonas hydrophila* AL09-71 | CP007566.1\|:639696-641732 |
| *Aeromonas salmonicida* 449 | CP000644.1\|:4068209-4070245 |
| *Aeromonas veronii* B565 | CP002607.1\|:3258161-3260200 |
| *Burkholderia mallei* ATCC 10399 | CH899680.1\|:197030-199282 |
| *Burkholderia pseudomallei* K96243 | BX571965.1\|:8395-10668 |
| *Dickeya chrysanthemi* | L02214.1\|ERWOUTCM:1550-3688 |
| *Dickeya dadantii* 3937 | CP002038.1\|:2886708-2888798 |
| *Dickeya zeae* Ech1591 | CP001655.1\|:1474160-1476310 |
| *Erwinia pyrifoliae* Ejp617 | CP002124.1\|:358927-360864 |
| *Escherichia coli* 2534-86 (pETEC) | AFDS01000066.1\|:12873-14933 |
| *Escherichia coli* 3030-2 (pETEC) | AFDT01000052.1\|:15359-16930 |
| *Escherichia coli* BW2952 (K-12) | CP001396.1\|:3341556-3343508 |
| *Escherichia coli* CE10 (NMEC) | CP001396.1\|:3558481-3560541 |
| *Escherichia coli* EC958 (UPEC) | HG941718.1\|:3387777-3389837 |
| *Escherichia coli* G58-1 | AFDX01000036.1\|:16791-18743 |
| *Escherichia coli* H10407 (hETEC) | AY056599.1\|:3304-5154 |
| *Escherichia coli* LF82 (AIEC) | CU651637.1\|:3499486-3501438 |
| *Escherichia coli* MG1655 (K-12) | U00096.3\|:3456377-3458329 |
| *Escherichia coli* Nissle 1917 | CP007799.1\|:3438477-3440537 |
| *Escherichia coli* NRG 857C (AIEC) | CP001855.1\|:3110841-3112901 |
| *Escherichia coli* UMNF18 (pETEC) | AGTD01000001.1\|:3641361-3643421 |
| *Escherichia coli* UMNKK88 (pETEC) | CP002729.1\|:3590375-3592435 |
| *Escherichia coli* W3110 (K-12) | AP009048.1\|:4182087-4184039 |
| *Klebsiella oxytoca* HKOLP1 | CP004887.1\|:5022762-5024741 |
| *Klebsiella pneumoniae* ATCC BAA-2146 | CP006659.1\|:955146-957119 |
| *Legionella longbeachae* D-4968 | ACZG01000001.1\|:527152-529512 |
| *Pectobacterium carotovorum* | X70049.1\|:1020-2969 |
| *Pseudomonas aeruginosa* PA1 | CP004054.1\|:1076643-1078616 |
| *Pseudomonas putida* H8234 | CP005976.1\|:1106479-1108227 |
| *Shewanella amazonensis* SB2B | CP000507.1\|:198247-200358 |
| *Shewanella loihica* PV-4 | CP000606.1\|:4315828-4317951 |
| *Shewanella putrefaciens* 200 | CP002457.1\|:426297-428414 |
| *Vibrio cholerae* TRH7000 | L33796.1\|VIBEPSCN: 1176-3200 |
| *Vibrio vulnificus* | CP002469.1\|:3070180-3071679 |
| *Escherichia coli strain* ATCC 25922 16S rRNA | DQ360844.1:86278349 |

^a^pETEC: porcine-derived enterotoxigenic *Escherichia coli*; NMEC: neonatal meningitis *E. coli*; UPEC: uropathogenic *E. coli*; hETEC: human-derived enterotoxigenic *E. coli*; AIEC: adherent-invasive *E. coli*.
