# Supplementary material for: Relationship between Heat-Labile Enterotoxin Secretion Capacity and Virulence in Wild Type Porcine-Origin Enterotoxigenic Escherichia coli Strains
Source: PLoS One. 2015 Mar 13;10(3):e0117663. doi: 10.1371/journal.pone.0117663 (PMC4358887; doi:10.1371/journal.pone.0117663)
Supplement: S5 Table — (DOCX) [file pone.0117663.s011.docx]

**Table S5.** Genetic sequences of *gspE* homologs used for generating the Maximum Likelihood phylogenetic tree.^a^

| **Strain** | **GenBank Accession No.** |
| --- | --- |
| *Aeromonas hydrophila* AL09-71 | CP007566.1\|:641732-643237 |
| *Aeromonas salmonicida* 449 | CP000644.1\|:4070245-4071750 |
| *Aeromonas veronii* B565 | CP002607.1\|:3260200-3261705 |
| *Burkholderia mallei* ATCC 10399 | CH899680.1\|:195540-197033 |
| *Burkholderia pseudomallei* K96243 | BX571965.1\|:10665-12158 |
| *Dickeya chrysanthemi* | L02214.1\|ERWOUTCM:3685-5181 |
| *Dickeya dadantii* 3937 | CP002038.1\|:2885230-2886708 |
| *Dickeya zeae* Ech1591 | CP001655.1\|:1476307-1477803 |
| *Erwinia pyrifoliae* Ejp617 | CP002124.1\|:360864-362330 |
| *Escherichia coli* 2534-86 (pETEC) | AFDS01000066.1\|: 11380-12873 |
| *Escherichia coli* 3030-2 (pETEC) | AFDT01000006.1\|:81778-83163 |
| *Escherichia coli* BW2952 (K-12) | CP001396.1\|:3343518-3344999 |
| *Escherichia coli* CE10 (NMEC) | CP001396.1\|:3556988-3558481 |
| *Escherichia coli* EC958 (UPEC) | HG941718.1\|:3386284-3387777 |
| *Escherichia coli* G58-1 | AFDX01000036.1\|:18753-20234 |
| *Escherichia coli* H10407 (hETEC) | AY056599.1\|:5154-6647 |
| *Escherichia coli* LF82 (AIEC) | CU651637.1\|:3501448-3502929 |
| *Escherichia coli* MG1655 (K-12) | U00096.3\|:3458339-3459820 |
| *Escherichia coli* Nissle 1917 | CP007799.1\|:3819104-3820585 |
| *Escherichia coli* NRG 857C (AIEC) | CP001855.1\|:3109348-3110841 |
| *Escherichia coli* UMNF18 (pETEC) | AGTD01000001.1\|:3639868-3641361 |
| *Escherichia coli* UMNKK88 (pETEC) | CP002729.1\|:3588882-3590375 |
| *Escherichia coli* W3110 (K-12) | AP009048.1\|:4180596-4182077 |
| *Klebsiella oxytoca* HKOLP1 | CP004887.1\|:5021257-5022762 |
| *Klebsiella pneumoniae* ATCC BAA-2146 | CP006659.1\|:953656-955149 |
| *Legionella longbeachae* D-4968 | ACZG01000001.1\|:529512-530999 |
| *Pectobacterium carotovorum* | X70049.1\|:2966-4462 |
| *Pseudomonas aeruginosa* PA1 | CP004054.1\|:1074203-1075711 |
| *Pseudomonas putida* H8234 | CP005976.1\|:1108227-1109675 |
| *Shewanella amazonensis* SB2B | CP000507.1\|:200369-201931 |
| *Shewanella loihica* PV-4 | CP000606.1\|:4314264-4315835 |
| *Shewanella putrefaciens* 200 | CP002457.1\|:428407-429972 |
| *Vibrio cholerae* TRH7000 | L33796.1\|VIBEPSCN:3197-4708 |
| *Vibrio vulnificus* | CP002469.1\|:3068963-3070180 |
| *Escherichia coli strain* ATCC 25922 16S rRNA | DQ360844.1:86278349 |

^a^pETEC: porcine-derived enterotoxigenic *Escherichia coli*; NMEC: neonatal meningitis *E. coli*; UPEC: uropathogenic *E. coli*; hETEC: human-derived enterotoxigenic *E. coli*; AIEC: adherent-invasive *E. coli*.
